# Metronomic Photodynamic Therapy with Conjugated Polymer Nanoparticles in Glioblastoma Tumor Microenvironment

**DOI:** 10.3390/cells12111541

**Published:** 2023-06-04

**Authors:** Matías Daniel Caverzán, Paula Martina Oliveda, Lucía Beaugé, Rodrigo Emiliano Palacios, Carlos Alberto Chesta, Luis Exequiel Ibarra

**Affiliations:** 1 Instituto de Investigaciones en Tecnologías Energéticas y Materiales Avanzados (IITEMA), Universidad Nacional de Río Cuarto (UNRC) y Consejo Nacional de Investigaciones Científicas y Técnicas (CONICET), Río Cuarto X5800BIA, Argentina; 2 Departamento de Patología Animal, Facultad de Agronomía y Veterinaria, Universidad Nacional de Río Cuarto, Río Cuarto X5800BIA, Argentina; 3 Departamento de Biología Molecular, Facultad de Ciencias Exactas, Fisicoquímicas y Naturales, UNRC, Río Cuarto X5800BIA, Argentina; 4 Instituto de Biotecnología Ambiental y Salud (INBIAS), Universidad Nacional de Río Cuarto (UNRC) y Consejo Nacional de Investigaciones Científicas y Técnicas (CONICET), Río Cuarto X5800BIA, Argentina; 5 Departamento de Química, Facultad de Ciencias Exactas, Fisicoquímicas y Naturales, UNRC, Río Cuarto X5800BIA, Argentina

**Keywords:** conjugated polymer nanoparticles, metronomic photodynamic therapy, tumor-associated macrophages, glioblastoma

## Abstract

Alternative therapies such as photodynamic therapy (PDT) that combine light, oxygen and photosensitizers (PSs) have been proposed for glioblastoma (GBM) management to overcome conventional treatment issues. An important disadvantage of PDT using a high light irradiance (fluence rate) (cPDT) is the abrupt oxygen consumption that leads to resistance to the treatment. PDT metronomic regimens (mPDT) involving administering light at a low irradiation intensity over a relatively long period of time could be an alternative to circumvent the limitations of conventional PDT protocols. The main objective of the present work was to compare the effectiveness of PDT with an advanced PS based on conjugated polymer nanoparticles (CPN) developed by our group in two irradiation modalities: cPDT and mPDT. The in vitro evaluation was carried out based on cell viability, the impact on the macrophage population of the tumor microenvironment in co-culture conditions and the modulation of HIF-1α as an indirect indicator of oxygen consumption. mPDT regimens with CPNs resulted in more effective cell death, a lower activation of molecular pathways of therapeutic resistance and macrophage polarization towards an antitumoral phenotype. Additionally, mPDT was tested in a GBM heterotopic mouse model, confirming its good performance with promising tumor growth inhibition and apoptotic cell death induction.

## 1. Introduction

In recent decades, interest in photodynamic therapy (PDT) against brain tumors has increased considerably [1,2,3,4]. Brain tumors, especially malignant gliomas such as glioblastomas (GBMs), require alternative therapeutic options to increase the poor survival rate after diagnosis. Given the spatial selectivity of photo-assisted therapies and their compatibility with other therapeutic options, PDT has been proposed as either the main therapy or an adjuvant therapy for the treatment of many solid tumors including GBM [5,6]. Photodynamic techniques such as photodynamic diagnosis (PDD), fluorescence-guided tumor resection (FGR) and PDT have been continuously studied in clinical trials as adjuvant treatments for malignant brain tumors [7,8].

PDT is a non-invasive therapeutic procedure based on the light-triggered reaction of a photosensitizer (PS) with triplet oxygen (^3^O_2_), which leads to the generation of reactive oxygen species (ROS). After photoactivation, the PS initially reaches an excited singlet state (^1^PS*), which can evolve into an excited triplet state (^3^PS*) by intersystem crossing. ^3^PS* is an energetic specie that usually reacts following two main pathways leading to oxidation [9]: (1) type I reactions, which involve an electron transfer process to ground-state molecular oxygen (^3^O_2_) and other substrates, eventually producing superoxide anions (O_2_˙^−^), hydroxyl radicals (OH˙) and hydrogen peroxide (H_2_O_2_); or (2) type II reactions, where singlet oxygen (^1^O_2_) is generated as a result of energy transfer from ^3^PS* to ^3^O_2_. The type II mechanism has been frequently postulated as a prevalent and desired process to induce damage in tumor cells [10,11]. The localized action of PDT is due to the combination of the precise spatial control of illumination and the relatively short lifetime of ^1^O_2_ (i.e., <4.0 μs) [12] and other ROS that result in short diffusion distances of the reactive species [13]. This confined action is required to produce highly localized damage to tumor cells while minimizing the risk of damage to adjacent normal brain tissue, which is a limitation for classical adjuvant treatments to surgery [14].

The design of reliable clinical protocols requires careful consideration of not only the basic PDT ingredients (excitation wavelength, PS nature and the presence of ^3^O_2_) but also of careful PS and light dosimetry [15]. Increases in the light dose and PS concentration usually lead to better PDT efficiency in oxygen-saturated conditions [16]. However, it is well known that GBM, such as other cancer types, are hypoxic in some regions [17,18], leading to a limited supply of oxygen from the vasculature and tumor surrounding for the PDT photochemical reactions. In clinical settings, PDT is prescribed taking into account the PS concentration and the light dose. The latter depends on the applied light fluence rate or irradiance (mW/cm^2^) and the fluence of the total light dose (J/cm^2^), which involves the irradiation time as well (total energy delivered within a specific light time interval) [9,19]. Conventional PDT (cPDT) requires a light source capable of delivering irradiance values ≥100 mW/cm^2^. PDT protocols using these high photon flux density levels can lead to therapeutic complications such as organ damage and post-operative adhesion [20]. An additional important disadvantage of PDT protocols using high light fluence rates is the abrupt consumption of oxygen, which leads to a decrease in the efficacy of cPDT [21,22]. In addition, various cell-dependent factors triggered by PDT, depending on the intensity and duration of the treatment, have been postulated as resistant cellular mechanisms, including hypoxia and ROS-related survival pathways such as HIF-1 signaling [16,23,24]. The activation of HIF-1 by hypoxia and/or high levels of ROS triggers the transcription of hypoxia-responsive genes that have been associated with a genetic and epigenetic adaptation of tumor cells promoting tumor progression events and also PDT therapeutic resistance [3,25].

Conventional metronomic regimens are based on the administration of drugs at continuous intervals and/or at low and non-toxic doses, which results in longer treatment periods than conventional regimens [26]. Regarding PDT, the metronomic regimen (mPDT) could be achieved by the administration of low PS doses as well as by the administration of low light irradiance and for extended time periods to increase the selectivity of the therapy and minimize the development of therapy resistance. Efforts were made in the past to evaluate this type of modality with classical molecular PSs such as 5-aminolevulinic acid (ALA) and photofrin [20,27,28]. Regarding malignant brain tumors, mPDT was used in some studies and was aimed mainly at the selective destruction of tumor cells versus normal brain tissue. Bisland S., Wilson B. and colleagues demonstrated for the first time that mPDT using molecular PSs induces a pronounced photokilling action in preclinical brain tumor animal models [29,30]. However, to date, there have been no studies investigating mPDT with third-generation PSs such as conjugated polymer nanoparticles (CPNs). This type of nanoparticulated PS has been studied for the PDT of several cancer types and was shown to efficiently generate ^1^O_2_ [1,16,31,32]. CPNs have several advantages compared to molecular PSs, such as superb extinction coefficients (several orders of magnitude higher than molecular PSs), excellent photostability and easy surface functionalization with bioactive molecules for cell targeting [33,34,35]. Therefore, in this study, we evaluated mPDT using CPNs composed of poly(9,9-dioctylfluorene-alt-benzothiadiazole) F8BT and poly(styrene-co-maleic anhydride) (PSMA) and doped with platinum porphyrin (PtOEP); the particles were termed CPNs. We compared the in vitro effectiveness of mPDT vs. cPDT, evaluating the GBM cell viability, the PDT impact on the modulation of the phenotypic profile of the main non-tumor cell population of the tumor microenvironment (TME) and HIF-1α activation as an indirect indicator of oxygen consumption. GBM TME is mainly represented by the presence of infiltrating tumor-associated macrophages (TAMs), which can occupy up to 50% of the tumor mass [36,37]. Therefore, an in vitro evaluation was performed in GBM monocultures of tumor cells and co-cultures with TAMs as well. Co-cultures represent more appropriated in vitro models to contemplate the spatial organization of cell–cell interactions and the subsequent response to new potential treatment modalities [38,39]. By using co-cultures, it is possible to recreate some of the in vivo GBM tumor niches, which allows for a more relevant therapeutic efficiency evaluation of both PDT modalities. In vitro experiments revealed a more pronounced cell death with less HIF-1 activation when using CPNs in mPDT vs. cPDT in monocultures of GBM cells. In addition, co-cultures of GBM and TAMs revealed a protective antitumoral effect of TAMs towards the CPN-PDT of GBM cells in both irradiation modalities, which denotes the importance of contemplating other non-tumor populations belonging to the TME in the preclinical evaluation of therapeutic compounds. However, mPDT irradiation continued to be more effective than the conventional modality. Finally, mPDT with CPNs was validated in a xenograft GBM mouse model, showing a significant antiproliferative effect with a considerable impact on tumor growth and tissue morphological changes, confirming the good performance of metronomic regimens with nanoparticulated PSs.

## 2. Materials and Methods

### 2.1. Materials

The fluorescent polymer poly(9,9-dioctylfluorene-alt-benzothiadiazole) (F8BT, Mn = 70,000 g/mol, PDI = 2.4, American Dye Source, Baie-d’Urfé, QC, Canada), the amphiphilic functional polymer poly(styrene-co-maleic anhydride) (PSMA, terminated by cumene, content of 68% styrene, average molecular weight about 1700 g/mol, Sigma Aldrich, St. Louis, MO, USA), and the porphyrin Pt(II) octaethylporphyrin (PtOEP, >95%, Frontier Scientific, Logan, UT, USA), were used as received. To dissolve polymers, tetrahydrofuran (THF, pro-analysis grade, Sintorgan, Villa Martelli, Argentina) was used after reflux for 5 h with potassium hydroxide pellets (KOH, pro-analysis grade, Taurus). Nanoprecipitation was carried out in double-distilled water that was further purified by an ELGA PURELAB Classic UV system (~18.2 MΩ/cm) to remove ions and organic and particulate matter (0.2 μm pore diameter filter). Phorbol 12-myristate 13-acetate (PMA) (Sigma-Aldrich, St. Louis, MO, USA), 2′,7′-dichlorodihydrofluorescein diacetate (DCFH-DA, Merck, New York, NY, USA) and 3-(4,5-dimethylthiazol-2-yl)-2,5-diphenyl tetrazolium bromide) (MTT, Sigma, St. Louis, MO, USA) were used as received.

### 2.2. Nanoparticle Synthesis

CPNs were developed by a previously reported nanoprecipitation protocol [33]. Briefly, a stock solution of F8BT was prepared, dissolving the polymer in fresh THF to a concentration of ~500 mg/L. This solution was filtered with a 0.2 μm pore size PTFE membrane syringe filter (Iso-Disc, Sigma-Aldrich, St. Louis, MO, USA) to remove undissolved polymers. The concentration of the filtered solution was recalculated from its absorption spectrum using a known absorption coefficient (45.4 g^−1^ L cm^−1^ in THF at 456 nm) [40]. On the other hand, stock solutions of PSMA (2 g/L) and PtOEP (0.25 g/L) in THF were also prepared. Afterwards, all three solutions were mixed in THF to a final concentration of 50, 10 and 5 mg/L for F8BT, PSMA and PtOEP, respectively. A volume of 5 mL of F8BT/PSMA/PtOEP solution was quickly injected into 10 mL of milliQ water while sonicating (Arcano, PS-30A, Buenos Aires, Argentina), and the resulting mixture was further sonicated for 10 min. THF was removed under reduced pressure in a rotary evaporator, yielding a final volume of 10 mL. The injection procedure was repeated with a new F8BT/PSMA/PtOEP solution in THF (5 mL). Again, THF was completely removed, and water was partially removed on this occasion. Finally, the obtained nanoparticle dispersion was filtered through a 0.2 μm pore size cellulose acetate membrane filter (25 mm, gamma sterile, Micron Separation Inc., Westborough, MA, USA) to eliminate large aggregates. CPN concentration after filtering was expressed in terms of F8BT mass concentration (mg/L) and was calculated using the absorption coefficient of neat F8BT CPN [40]. Different stock batches of CPN suspensions were prepared with a final F8BT concentration of ~100 mg/L. CPNs were characterized by dynamic light scattering (DLS) and by absorption and emission spectra.

### 2.3. Cell Lines and Culture Conditions

Three human GBM tumor cell lines were used: U87-MG (ATCC^®^ HTB-14™), derived from a glioblastoma of an adult male patient; T98G (ATCC^®^ CRL-1690™), derived from a 61-year-old human male patient with glioblastoma and M059K (ATCC^®^ CRL-2365™), derived from a 33-year-old male patient with glioblastoma. Additionally, the human monocyte cell line THP-1 was used in co-cultures with GBM cells. U87-MG, T98G and MO59K were grown in Dulbecco’s modified eagle’s medium (DMEM, Sigma-Aldrich) supplemented with 10% fetal bovine serum (FBS, Internegocios, S.A, Buenos Aires, Argentina). THP-1 cells (ATCC^®^ TIB-202™) were grown in Roswell Park Memorial Institute-1640 medium (RPMI-1640, Sigma-Aldrich, St. Louis, MO, USA) supplemented with 10% FBS. All cells were maintained in a 5% CO_2_ atmosphere at 37 °C.

U87-MG and MO59K cells inducibly expressing hypoxia response element fused-green fluorescence protein (HRE-GFP) construct were generated by stable transfection with a plasmid containing the gene for EGFP placed under the control of a promoter region consisting of five copies of a 35-bp fragment from the HRE of the human *VEGF* gene. The plasmid was kindly provided by Dr. Foster (University of Rochester, Rochester, NY, USA). The transfection conditions were established previously [23,41]. Briefly, transfections were performed using X-tremeGENE™ HP DNA Transfection Reagent (Roche) according to the manufacturer’s instructions. U87MG-HRE- and MO59K-HRE-stable transfected cells were selected in growth medium supplemented with 400 μM of active Geneticin (G418) (Life Technologies). Individual colonies were isolated after 2–3 weeks of growth under selection using the cloning ring method and subsequently expanded into clonal cell lines.

On the other hand, U87-MG cells constitutively expressing red fluorescent protein RFP (U87MG-RFP) were generated by lentiviral infection of the pRFP-LC3 plasmid as previously described [2]. Expression of GFP or RFP was assessed by fluorescence microscopy and flow cytometry (FC).

For co-culture experiments, first THP-1 cells were seeded in a 24-well plate (30,000 and 50,000 cells/well) with the addition of PMA 50 ng/mL in RPMI completed medium. PMA was used to differentiate monocyte to macrophage phenotype and induce cell adhesion [2,42]. After 48 h of PMA induction, the medium was discarded, cells washed twice with PBS, and U87MG-RFP cells seeded over macrophages at a ratio of 2:1 (70,000 GBM cells/30,000 macrophages per well) and 1:1 (50,000 GBM cells/50,000 macrophages per well). These two GBM/macrophage ratios were chosen based on the evidence that macrophage could represent up to 30–50% of GBM tumor mass [43,44,45] and their abundance correlates with malignancy, tumor grade and therapeutic resistance [46,47].

### 2.4. Light Irradiation Device and Experimental Setup for PDT Experiments

A custom-designed multi light emitting diodes (LED) system equipped with 96 LED emitters (each LED having 1 W nominal electrical input and maximum wavelength of 460 ± 20 nm) was used for PDT experiments. The light output of the LEDs was adjustable by an electronic bench DC power supply. The irradiance (flux density) was measured at the sample plane in two different current and voltage settings using a Thorlabs PM100D power meter equipped with a Thorlabs S130C detector [48]. Measured irradiances were 12 mW/cm^2^ and 84 mW/cm^2^ for mPTD and cPDT configurations respectively. Light doses of 10 and 40 J/cm^2^ were used in both configurations. For the mPDT modality, the irradiation times necessary to reach 10 J/cm^2^ and 40 J/cm^2^ doses were 10 and 39 min, respectively. On the other hand, the radiant exposures were 2 and 8 min to reach 10 J/cm^2^ and 40 J/cm^2^, respectively, in cPDT configuration. For animal experiments, mPDT irradiation was implemented using a fiber optic coupled to the multi-LED system and controlled with the DC power supply. An irradiance of 12 mW/cm^2^ was achieved at the fiber exit end measured by Thorlabs PM100D and a radiant exposure time of 30 min was used.

### 2.5. PDT In Vitro of Monocultures of GBM Cell Lines

U87-MG, T98G and MO59K were seeded in 96-well plates (20,000 cells/well) and grown overnight at 37° in DMEM supplemented with 10% of FBS plus antibiotics. Then, medium was replaced with DMEM supplemented with 10% of FBS containing CPNs at different concentrations (6; 12; 24 and 48 mg/L) and cells were incubated for 24 h at 37 °C as previously described to allow cell incorporation [1,16]. Then, culture medium was replaced with fresh DMEM supplemented with 10% FBS. Afterwards, cells were irradiated in the two PDT modalities (cPDT and mPDT) and using total light doses of 10 and 40 J/cm^2^. Viability was examined 24 h after illumination using MTT assay as described previously and results were expressed as percentage relative to control cells (cells irradiated without CPNs) [1]. Three sets of independent experiments were performed (with *n* = 6 for each CPN concentration tested). Viability IC_50_ values were obtained by nonlinear regression fitting to the MTT results with GraphPad Prism 8software (version 8.2.1).

### 2.6. ROS Production Measurement after PDT

Intracellular ROS after PDT treatments were detected using the DCFH-DA fluorescent probe. GBM cells were cultured in 24-well plate (50,000 cell/well) and treated as follows. Cells were exposed to different CPN concentrations (6 and 12 mg/L) and were incubated for 24 h at 37 °C as previously described to allow particle incorporation. Then, cells were washed twice with PBS and incubated with 10 µM DCFH-DA in PBS at 37 °C for 30 min. Subsequently, medium was renewed, and cells were photoirradiated at the two modalities. The 2′,7′-dichlorofluorescin (DCF) fluorescence was recorded immediately after PDT treatment by flow cytometry [16,49]. To this purpose, cells were harvested with trypsin and resuspended in PBS for FC analysis. A total of 10,000 cells per sample were evaluated using a Guava easyCyte 6-2L flow cytometer with excitation and emission at 488 nm and 525/30 nm, respectively. Growing cells without any treatment were used as negative control and also cells exposed to equal CPN concentrations without irradiation were used to correct for intrinsic CPN green fluorescence [16]. A positive control of cells exposed to 10 mM H_2_O_2_ was also included. The mean fluorescent intensity in each treatment group was normalized to the mean fluorescent intensity of the control group for each cell line.

### 2.7. HIF-1 Activation Assay after PDT

The GBM U87MG-HRE and MO59K-HRE cell lines were used to evaluate HIF-1 activation upon application of both PDT modalities. To accomplish this, cells were cultured in a 24-well plate (100,000 cells/well) and exposed to 3 or 6 mg/L CPNs for 24 h. These concentrations were chosen, since it was previously determined that, in both PDT modalities, they induce the death of 50% of the cells, leaving viable cells to trigger the biological event of binding of HIF-1 to HRE and the transcription of GFP. After CPN incubation, cells were washed twice with PBS, renewed medium and treated with cPDT or mPDT (10 J/cm^2^). GFP fluorescence was recorded in FC instrument after cell detachment at 6 and 24 h post PDT. COCl_2_ (400 μM) was used as a positive control, since it inhibits prolylhydroxylases enzymes responsible for initiating the destabilization and destruction of HIF-1 by proteasomes [41].

### 2.8. Evaluation of Cell Death Mechanism

Apoptosis and necrosis events were evaluated using Annexin V-FITC/Propidium iodide staining kit II (BD Pharmingen™, New Jersey, USA) following the manufacturer’s instructions. U87-MG and T98G cells were used as the two cell lines with different therapeutic susceptibility. Cells (10,000 cells per well) were seeded into 24-well plates overnight. Then, cells were incubated with different CPN concentrations (6, 12 and 24 mg/L) for 24 h and irradiated with 10 J/cm^2^ in both modalities as described above. Three negative control experiments were performed: cells not exposed to CPNs and not irradiated, cells exposed to CPNs and not irradiated and cells irradiated using both PDT modalities not exposed to CPNs. Two positive controls were used: cells treated with heat (65 °C for 15 min) and cells exposed to doxorubicin (10 μM for 24 h). These controls were used to delimit the corresponding quadrants. The samples were finally analyzed at 6 h and 24 h after PDT on a Guava easyCyte 6-2L cytometer and data were processed with FlowJo software (version X 10.0.7r2). Two sets of independent experiments were performed (with *n* = 3 for each experiment).

### 2.9. PDT In Vitro Evaluation in Co-Cultures of GBM-TAMs

To evaluate mPDT in GBM TME, different co-culture in vitro models were established. The experimental conditions to develop these co-cultures are described in Section 2.1. When co-cultures of U87-MG- and THP-1-derived macrophages were established, cells were exposed to different CPN concentrations for 24 h. Later on, cultures were washed twice with PBS, and fresh medium was added to perform light irradiation. For both PDT modalities, a total dose of 10 J/cm^2^ was administered. The PDT effect was evaluated 24 h after treatment by determining cell viability by flow cytometry and using a LIVE/DEAD™ Fixable Far Red staining kit (Invitrogen, Waltham, MA, USA) as previously described [50] and by observation of the cells’ morphological changes (using brightfield and fluorescence microscopy in a Nikon Eclipse Ti-S microscope equipped with an LWD 20×/0.4 objective lens and a Nikon DS-QiMC CCD camera).

### 2.10. Gene Expression Analysis in TAMs of Co-Cultures after PDT

In order to evaluate polarization profile of THP-1-derived macrophages in co-culture conditions and after PDT treatments, THP-1 were seeded in 24-well plates (50,000 cells/well) with PMA 50 ng/mL in RPMI-completed medium. After 48 h incubation time, the medium was removed, the cells were washed three times with PBS and fresh RPMI-completed medium was added, and the samples were allowed a 24 h resting incubation period [42,51]. On the other hand, conditioned media (CM) were obtained from GBM U87-MG cells seeded in 6-well plates (200,000 cells/well) and treated with either a cPDT or mPDT (10 J/cm^2^ dose) previous incubation with 6 mg/L CPN for 24 h, as described in Section 2.3. To this purpose, media from U87-MG monolayers were recovered after PDT, clarified by centrifugation (10 min, 10,000 rpm) and stored at −80 °C until use [2]. Additionally, CM was obtained from GBM U87-MG-grown monolayers without PDT treatment.

Resting THP-1-derived macrophages were exposed to different CM treatments for 24 h. Afterward, RNA was extracted using TRIzol reagent (Invitrogen, Thermo Fisher Scientific, Waltham, MA, USA) and reverse transcribed using M-MLV reverse transcriptase (Invitrogen, Thermo Fisher Scientific) according to the manufacturer’s instructions. Quantitative PCR (RT-qPCR) was performed with SYBR Green qPCR Master Mix (Agilent Technologies, Santa Clara, CA, USA) and 10 ng of cDNA per reaction using Agilent’s Stratagene Mx3000PRO system [2]. GAPDH gene expression was used as a housekeeping gene to normalize the expression of target genes, and the 2^−ΔΔCT^ method was used to calculate the relative levels of gene expression using the Strata gene MxPro QPCR software v3.00 tool (Stratagene, Agilent Technologies). A standard melting curve cycle was used to confirm the quality of amplifi- cation. Each sample was analyzed in triplicate, and the experiment was repeated 3 times. Results were expressed as a relative fold of stimulation over the control group (resting THP-1-derived macrophages exposed to RPMI medium 10% FBS). C-C chemokine receptor type 7 (*CCR7*) and tumor necrosis factor alpha (*TNFα*) gene expressions were used to identify M1 macrophages. On the other hand, mannose receptor c-type 1 (*MCR-1*) and vascular endothelial growth factor A (*VEGFA*) were used to identify M2 macrophages [2,52,53]. Forward and reverse primers for the genes of interest are shown in Table 1.

### 2.11. GBM Xenograft Mouse Model

Immunodeficient BALB/c nude (nu/nu) adult male mice (~30 g) were housed in a humidity- and temperature-controlled room on a 12 h light/dark cycle, receiving water and food ad libitum and maintained under specific pathogen-free conditions. Experiments were in compliance with the Guide for the Care and Use of Laboratory Animals published by the NIH and approved by the Comité de Ética de la Investigación (COEDI) from Universidad Nacional de Rio Cuarto, Río Cuarto, Argentina (Cod. 300/21). The xenograft GBM model was implemented as follows: Briefly, animals were anesthetized by inhalation of 3% isoflurane in 99.9% O_2_ (1 L min^−1^) in an induction chamber and maintained at 1.5% isoflurane in 99.9% O_2_ (1 L min^−1^), which was delivered through a nose mask. Then, U87-MG cells were subcutaneously inoculated into the dorsal flanks of mice (2 × 10^6^ cells/0.1 mL/mouse). Tumor diameters were measured once a week using a caliper, and PDT treatment was assayed when the tumor volume reached approximately 150 mm^3^.

### 2.12. PDT In Vivo Evaluation

To evaluate CPN-mPDT efficacy in vivo, xenograft GBMs were generated in the flanks of athymic mice by subcutaneous injection of U87-MG cells. Experiments were performed on day 15 after implantation. Tumors of the same uniform size were employed (0.3–0.5 cm diameter), and animals were divided into different treatments and control groups by simple randomization (*n* = 5). CPNs were administered by intravenous (i.v.) and intratumoral (i.t.) routes. For i.v. injection, a 0.4 mg/kg CPN dose was used after anaesthesia of the animals with isoflurane 1.5%. The dose was achieved by injection of 200 μL of CPN solution (50 mg/L in PBS) into the caudal tail vein. For i.t. administration, a 0.1 mg/kg CPN dose was administered by injecting 50 μL of CPN solution in PBS. Light irradiation was performed 24 h after i.v. injection or 4 h after i.t. injection. Based on our previous results of biodistribution with similar CPNs, these times are the most indicated to allow penetration of nanoparticles into the GBM heterotopic tumors [54]. mPDT irradiation was implemented using a system of a fiber optic coupled to a multi-LED with a measured irradiance of 12 mW/cm^2^ (at the fiber exit end) and a radiant exposure time of 30 min. This procedure was carried out with the animals anesthetized with isoflurane 1.5% using a nose mask. The measurement of tumor volume (TV) by caliper was employed to evaluate the delay in tumor growth induced by CPN-mPDT treatment. The longer and shorter perpendicular axes of each tumor at the initial day (day 0) were used to calculate TV and to normalize the following measurements up to a period of 10 days from the day of PDT application. TV was calculated by the following modified ellipsoidal formula [55]:

TV = 0.5 × long diameter × (short diameter)^2^ (1)

where the long diameter is the maximum distance between the outer tumor margins, expressed in mm, and the short diameter is the distance between the inner and outer tumor margins, expressed in mm.

In addition, we also evaluated histopathological changes in tumor tissues and other organs after the procedure (12 days after PDT treatment).

### 2.13. Statistical Analysis

Data are shown as mean values ± standard deviation of three independent experiments. Statistical analyses were performed using the GraphPad Prism software version 8.0 (GraphPad Software, San Diego, CA, USA). Kolmogorov–Smirnov normality tests were applied to further compare unpaired groups. According to the design of the experiment under analysis, the comparison of means between separate groups of numerical variables was performed using a one-way or two-way analysis of variance (ANOVA) and Tukey´s test. Nonlinear regression analysis was performed with the GraphPad statistical program using the cytotoxicity (%) values. With this analysis, the IC_50_ (dose leading to death of 50% of the current cell population) values on the cell lines after PDT were determined. The statistical significance threshold was set at *p* < 0.05.

## 3. Results and Discussion

### 3.1. Synthesis of CPNs

CPNs were prepared using a nanoprecipitacion method as previously reported by our group [1,16,33,48]. F8BT and PSMA polymers constituted the main components of the CPNs, producing stable suspensions in water for several months. PtOEP was incorporated in order to increase ^1^O_2_ production after light irradiation [10]. Dynamic light scattering measurements were performed to assess the hydrodynamic diameter of the CPNs (Figure S1). Average values of ~18 nm were obtained for these nanoparticles with a polydispersity index (PDI) of 0.12. The size distribution of the nanoparticle samples was related to the PDI value, where the PDI value and the size homogenization were inversely proportional. The addition of the amphipathic copolymer PSMA was crucial to preserve the colloidal stability of the particles in the culture medium. PSMA has statistically arranged styrene and maleic anhidride groups, which are thought to self-assemble into CPN structures by intercalating the planar styrene rings into the CP core structure. The maleic anhidride groups hydrolyze in water, producing maleic acid groups that are deprotonated at near neutral pH. These groups are presumably located on the CPN surface, and the resulting negative charges increase the particle colloidal stability in high-ionic-strength solutions such as buffers and culture media. The incorporation of PSMA in the sinthesis of CPNs has been reported to increase the zeta potential (ζ = −45 mV) and to reduce the averange size distribution of the resulting particles as compared to CPNs prepared using polystyrene grafted with ethylene oxide (PS-PEG-COOH) as the amphipilic polymer (Figure S1) [1,10]. The hydrodynamic size and spectroscopic data of the CPNs prepared in this work were in line with previously reported results [33]. The spectra characterization of the CPNs stabilized with PSMA in water, PtOEP in deoxygenated THF and CPNs-PSMA-PtOEP in water are shown in Figure S1.

### 3.2. PDT Cytotoxicity in Monocultures of GBM Cell Lines

In order to compare the therapeutic efficacy of the mPDT vs. cPDT irradiation modalities, monocultures of GBM cells (U87-MG, MO59K and T98G) were cultured in 96-well plates for subsequent experiments. These cell lines have been used extensively as in vitro cellular models of GBM, as they partly represent the intertumoral heterogeneity that this disease possesses. For instance, T98G has a mutated *TP53* gene, which is one of the most deregulated genes among different types of tumors and is particularly deregulated in 84% of GBM patients and in several GBM cell lines [56]. Deregulated components of the p53 pathway have been implicated in GBM cell invasion, migration, proliferation and evasion of apoptosis as well as in conventional chemoresistance [56,57,58]. Furthermore, we have previously reported the differential antioxidant capacity possessed by T98G cells with a negative impact on PDT efficacy [16].

**Figure 1:**
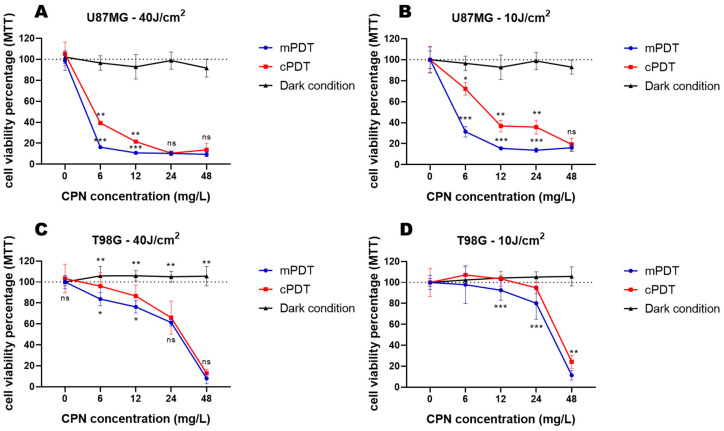
Quantification of cell viability by MTT assay 24 h after PDT for U87-MG (**A**,**B**) and T98G (**C**,**D**) cell lines as a function of CPN concentration during incubation and light doses using both irradiance fluence density corresponding to mPDT (blue lines) and cPDT (red lines). Incubation with CPNs in the dark is represented by black lines. Cell viability percentages were normalized to control cells exposed to light irradiation only. * *p* < 0.05, ** *p* < 0.01 and *** *p* < 0.001 with ANOVA and Tukey’s tests; ns: no statistically significant differences.

In the present study, light doses of 10 and 40 J/cm^2^ were delivered using irradiance values corresponding to the mPDT and cPDT modalities. To strictly compare these irradiation modalities, a common protocol was followed for all the GBM cell lines. Cells not incubated with CPNs were viable at all the light doses employed in the two modalities, whereas a significant toxic effect was found in all the cell lines after PDT. Figure 1 shows the viability of the studied cell lines incubated with various concentrations of CPNs and irradiated with different light doses in the different modalities. The U87-MG cells were the most sensitive to the cytotoxic effect of PDT in both radiant exposure dosimetries and power density modalities (Figure 1A,B). The mPDT application resulted in a significant decreased cell viability even at the lower CPN concentration tested of 6 mg/L (*p* ≤ 0.001) in this GBM cell line. As can be seen, a light dose of 10 J/cm^2^ (Figure 1B) in combination with increasing CPN concentrations was very effective in inducing cell death, as was 40 J/cm^2^ (Figure 1A) at the same irradiation modalities. For instance, the same CPN concentration (6 mg/L) at 10 J/cm^2^ induced a decrement in cell viability of 31.4 ± 5% using the mPDT modality compared to the 72.3 ± 6% cell viability in the conventional regimen. At a superior light dose regiment (40 J/cm^2^), the concentration of 6 mg/L drastically decreased the cell viability to 16.3 ± 1.6% in the mPDT modality in comparison with the conventional regimen where the cell viability was 39.4 ± 1%. These experiments at equal CPN concentrations but with changing light irradiances (mW/cm^2^) to achieve the same total light doses demonstrated a significant increased outcome using the mPDT modality. The calculated half maximal inhibitory concentration (IC_50_) also showed that mPDT (IC_50_ = 0.8 and 4.5 mg/L for 40 and 10 J/cm^2^, respectively) was more effective compared to cPDT (IC_50_ = 3.5 and 11.3 mg/L for 40 and 10 J/cm^2^, respectively). The other cell lines also had a similar behavior regarding the improved efficiency of mPDT, and the calculated IC_50_ values were lower for mPDT compared to cPDT in the T98G and MO59K cells (Table 2). T98G was the most resistant cell line to PDT cytotoxicity (Figure 1C,D). However, mPDT significantly decreased the cell viability compared to cPDT, mainly at the lowest CPN concentrations (*p* ≤ 0.001). In fact, the radiant exposure of 10 J/cm^2^ seemed to be the most appropriate light dosimetry at higher CPN concentrations, which suggested that a metronomic regimen could enhance therapeutic efficacy even in resistant GBM cell lines. MO59K had a similar behavior to U87-MG (Figure S2), which was in concordance with previous results from our group with other CPNs [16].

It is worth noting that PDT cytotoxicity was superior in the GBM cell lines using CPN-PSMA-PtOEP compared with our previous study with PtOEP-doped CPN using PS-PEG-COOH as a stabilizer [16]. Therefore, cell uptake was evaluated using both types of CPNs at equal concentrations in order to compare them in terms of their binging ability. We observed a greater cell uptake in all the GBM cell lines with CPN-PSMA-PtOEP (Figure S3), which are slightly smaller nanoparticles that have a more negative surface charge than CPN-PSPEG-PtOEP. A similar uptake result was previously obtained in breast cancer cell lines [33].

### 3.3. PDT Effects on HIF-1 Activation and ROS Production in GBM Cells

**Figure 2:**
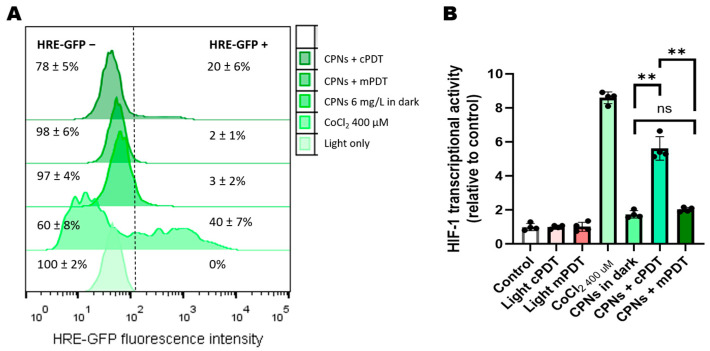
HIF-1 transcriptional activity. (**A**) Representative FC histograms of GFP expression; (**B**) HIF transcriptional activity of HIF + cells is indicated by GFP intensity fold increment (MFI, arbitrary units, A.U., compared with control cells) and refers to non-treated conditions. ** *p* < 0.01 with ANOVA and Tukey’s tests; ns: no statistically significant differences.

To assess the impact of both modalities (mPDT vs. cPDT) on HIF-1 transcriptional activity as an indirect indicator of intracellular oxygen consumption, the GBM cell lines U87MG-HRE and MO59K-HRE that were genetically modified to express GFP-associated HRE were used. Figure 2 shows the results obtained by FC. Treatment with CoCl_2_ (400 μM) induced HIF-1 activation in 40% ± 7 of the U87-MG-HRE cells. CoCl_2_ inhibits PHDs, which are the enzymes responsible for initiating the destabilization and destruction of HIF-1 by proteasomes. For this reason, CoCl_2_ is used as a hypoxia mimetic and produces the consequent stabilization of HIF-1 [23]. Neither the treatments with a concentration of 6 mg/L CPN in dark conditions or the treatments with irradiation in both modalities by themselves induced a significant activation of HIF-1 (Figure 2B).

When the PDT with CPN combination was assayed, a 2% ± 1 activation of HIF-1 was observed in the cells treated with 6 mg/L of CPN and irradiated with 10 J/cm^2^ in the mPDT modality. On the contrary, cPDT with an equal CPN concentration (6 mg/L) and irradiated with 10 J/cm^2^ induced HIF-1 activation in 20% ± 6% of the U87-MG cells (Figure 2A). MO59K-HRE showed a similar behavior. These results suggested that the cPDT modality compared to the mPDT modality consumed more intracellular oxygen as a result of the photochemical reactions that generated ROS at equal CPN concentrations.

In addition, the mean fluorescence intensity (MFI) of the HRE-GFP-positive cells in the different treatment groups was analyzed as a quantitative measurement of HIF transcriptional activity (Figure 2B). MFI values were expressed relative to the control group values, and they were lower in the mPDT group compared to the cPDT group. These differences were statistically significant (*p* < 0.01).

**Figure 3:**
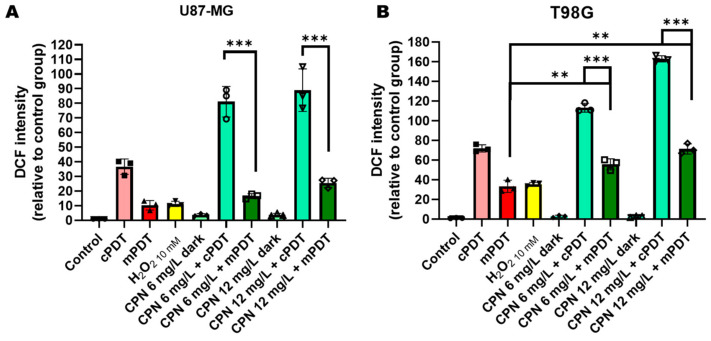
ROS production evaluation after PDT. Geometric mean fluorescence intensity quantification relative to autofluorescence of control group for U87-MG (**A**) and T98G (**B**). ROS levels were determined immediately after each treatment with DCFDA assay using flow cytometry. ** *p* < 0.01, *** *p <* 0.001 with ANOVA and Tukey’s tests.

To evaluate the importance of ROS-mediated damage induced by both modalities of CPN-PDT in the GBM cells, ROS levels were quantified immediately after the treatments using FC with a DCFDA probe. As shown in Figure 3, PDT-induced DCF oxidation was higher with cPDT irradiation compared to with mPDT even with only light irradiation. This result suggested that higher light fluence rates were highly stressful for the U87-MG and T98G GBM cells, as can be seen in the groups of cells irradiated and not exposed to CPNs (Figure 3). Additionally, cPDT with increased CPN concentrations (6 and 12 mg/L) further increased the DCF fluorescence intensity, which could be related to intense ROS production, leading to an abrupt oxygen consumption that was self-limiting for PDT [59,60]. In this case, an increased ROS production level was not associated with a superior cell death at these CPN concentrations for cPDT compared with mPDT (Figure 1). Elevated levels of ROS generated by PDT have previously been associated with HIF-1 activation [23]. Therefore, conventional PDT modalities with high light fluence rates would not only be self-limiting for photodamaging reactions but could also have a negative impact through the activation of molecular therapeutic resistance pathways. On the other hand, mPDT with increased CPN concentrations (6 and 12 mg/L) significantly elevated intracellular ROS levels compared with light irradiation only and with CPN incubation in dark conditions. The superior cell death in this irradiation modality could be associated with persistent oxidative damage due to the longer lighting time required in this modality. The T98G cells were the most stressed cells, however this cell line had more significant antioxidant mechanisms that favored its resistance to PDT [16].

### 3.4. Cell Death Mechanism Evaluation after PDT

**Figure 4:**
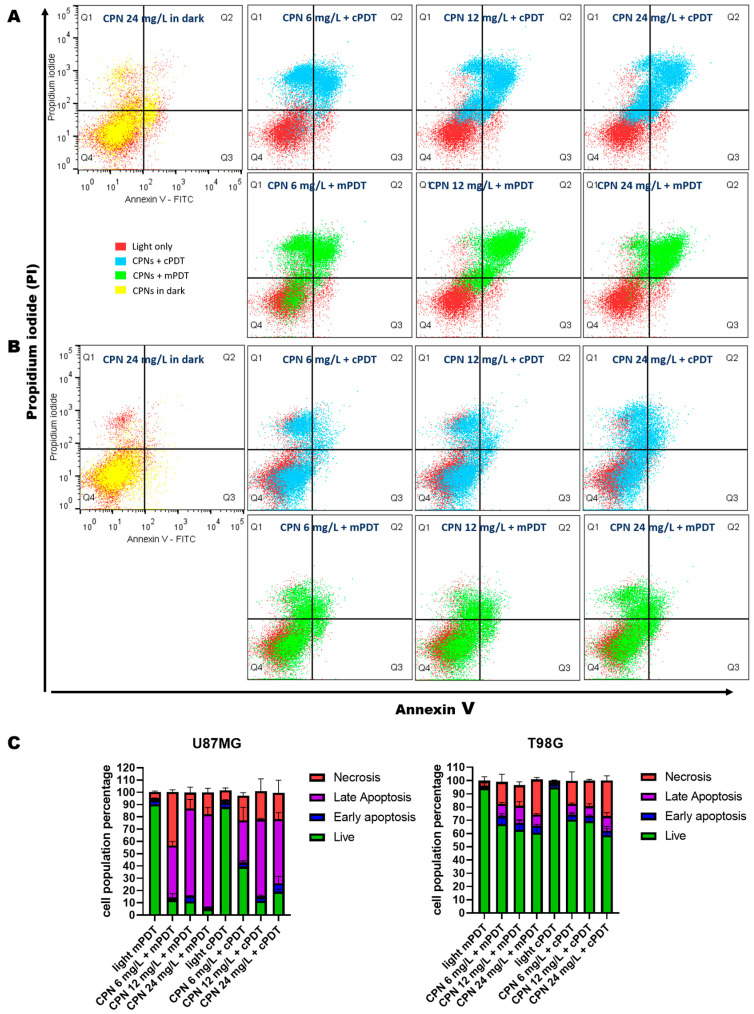
Cell death mechanism evaluation by FC. Dot plots graphs of Annexin V/PI-stained U87-MG cells (**A**) and T98G cells (**B**) treated with different CPN-PSMA-PtOEP concentrations (6, 12 and 24 mg/L) for 24 h and irradiated with 10 J/cm^2^ of mPDT (green dots) or cPDT (light blue dots) modalities compared to irradiation with light alone (red dots). The graphs with yellow dots represent the incubation with CPNs in the maximum concentration tested under non-irradiation conditions. (**C**) Quantitative analysis of the live, apoptotic and necrotic cells after PDT of U87-MG and T98G with mPDT or cPDT at 10 J/cm^2^.

The mechanisms of cell death induced by both PDT modalities were tested using a FITC Annexin V Apoptosis Detection Kit II, as described in the experimental section. Differences between the types of cell death resulting from the irradiation modalities have previously been reported [61]. The double staining of cells with FITC and PI monitored by FC allowed the discrimination of life (FITC−/PI−), early apoptosis (FITC+/PI−), late apoptosis (FITC+/PI−) and necrosis (FITC−/PI+). As shown in Figure 4, controlled light irradiation in both modalities (red dots groups in dot-plot graphs) did not induce cell death (number of cells alive above 90%), nor did CPN incubation without irradiation. On the contrary, the cells incubated with increasing CPN concentrations and irradiated in the mPDT or cPDT modalities at 10 J/cm^2^ were stained with Annexin V-FITC and PI in different proportions, which suggested variable degrees and types of cell death (Figure 4A,B). For the T98G cells, the predominant mechanism of PDT-induced death was necrosis, and significant percentages of apoptotic cells were observed with the mPDT modality in the three CPN concentration tested (Figure 4C). On the other hand, the U87-MG cells showed a larger fraction of late apoptotic cells as the main cell death mechanism at 6 h post PDT; necrosis was also evident in both PDT irradiation modalities. During the initial phase of apoptosis, phosphatidylserine in the cell membrane underwent translocation from the inner to the outer leaflet. As apoptosis progressed to the late stage, the cells exhibited an increased membrane permeability, allowing PI to enter and stain the DNA, eventually leading to the disintegration of the cellular structures and the formation of apoptotic bodies. The Annexin V/PI results were in agreement with the MTT results (vide supra), where the viable cell percentages were similar to those obtained with the colorimetric assay.

The percentage of late apoptotic cells increased with the CPN concentration in the mPDT modality at 6 h post treatment compared to the cPDT modality. The latter induced more necrotic cell death than the mPDT modality at the same time. Therefore, we can suggest that the mPDT modality favored a type of cell death by apoptosis, while the conventional modality induced cell death by necrosis to a greater extent. ROS formed upon irradiation, such as ^1^O_2_, have a limited lifetime and ability to migrate from the site(s) of their formation [12]. Thus, they interact with biologic substrates in the sites of PS localization to trigger cell death. Effective PS localization in the mitochondria, endoplasmic reticulum (ER), Golgi apparatus, lysosomes and/or plasma membrane have been associated with apoptosis or necrosis cell death mechanisms as well as with other cell death mechanisms such as necroptosis or apoptosis [62,63]. Previous results by our group and others [1,64] have shown that the cellular localization of these type of CPNs is mainly in lysosomes, although other cell components such as the plasma membrane or ER were associated with CPN binding. A stress insult on these organelles can trigger cell apoptosis. However, it is known that the intensity of the insult could change the cell death mechanism [65], and for PDT regimens, modification of the PS concentration and/or light doses could switch the type of cell death [66]. The type of cell death could switch from apoptosis to necrosis with the increasing intensity of the insult [62]. In our case, we suggest that the different light dosimetry regimes at equal CPN concentrations produced different intracelullar ROS amounts, as can been seen in the DCF oxidation experiments. The mPDT regimen increased intracelullar ROS production in a more sustained manner over time, which was associated with a not-so-aggressive insult to organelles to trigger cell death by apoptosis. On the other hand, the cPDT regimen increased ROS production in a very abrupt way but in a shorter period of time, causing overwhelming photodynamic damage to the cells, leading to disruption of the structural integrity of the plasma membrane and necrosis. Previous studies using molecular PSs such as ALA showed a pronounced earlier and late apoptosis after mPDT than after cPDT and necrosis after cPDT that mPDT, which is consistent with our results [61,67].

### 3.5. In Vitro Evaluation of PDT in Co-Cultures of GBM-TAMs

Because in real conditions, tumor cells are not independent cells but rather coexist in a microenvironment that can induce changes in their phenotype and in their therapeutic response, we sought to evaluate the preferential uptake of CPN in co-culture conditions of tumor cells with macrophages and the response to both PDT modalities. Indeed, it has been suggested that cell responsiveness to PDT is different between cancer cells and normal cells, as the resistance of these cell types to ROS stress is not the same [35]. Therefore, we cultured and differentiated THP-1 cells to a macrophage phenotype with PMA for the subsequent seeding of +red fluorescent protein (RFP)-expressing U87-MG cells (U87MG-RFP). The identification of tumor cells vs. THP-1-derived macrophages was based on + RFP vs. non-staining cells in fluorescence microscopy and FC (Figure S4). It was possible to cultivate both cell lines under these conditions whilst conserving their characteristic cell morphologies and with a cell viability greater than 90% in both cell types (Figure S4). This labeled/non-labeled cells strategy was employed to assess the differential CPN uptake and determine the cytotoxicity in each of the resident populations in the co-cultures.

Both in vitro and in vivo studies have shown that TAMs accumulate within GBM and that they are educated to adopt tumor-friendly phenotypes [68]. In order to evaluate the TAM population phenotype in co-culture conditions with GBM cells, a phenotype characterization by gene markers was performed to evaluate the transcript expression of relevant markers, such as *TNF-a* and *CCR7* for M1 and *MCR-1* and *VEGFA* for M2 profiles, after GBM CM exposure in THP-1-derived macrophages. Gene expression for *TNF-a* and *CCR7* increased significantly after PMA maturation, but the expression of these genes decreased to a great extent during the following days after exposure with GBM CM (Figure S5). On the contrary, the transcript expression of *MCR-1* and *VEGFA* was approximately two and three times higher compared to the M0 macrophages. This result suggested that the macrophages exposed to the secretome of GBM tumor cells were polarized towards a pro-tumor profile. Similar findings have been previously reported [69,70].

**Figure 5:**
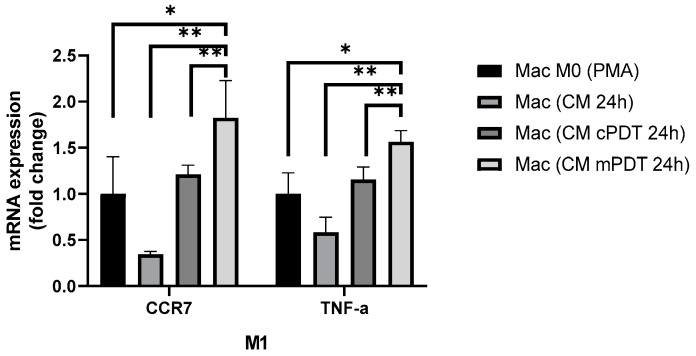
Gene expression of macrophage M1 phenotype evaluation after PDT. Mean relative gene expression of markers for M1 (*n* = 3 for each gene marker, mean ± SD) normalized to GADPH housekeeping and relative to THP-1-derived macrophages after PMA differentiation protocol and resting period of 24 h in complete growth medium. * *p* < 0.05; ** *p* < 0.01.

On the other hand, M1 markers were overexpressed in the macrophages exposed to CM from GBM cells treated with PDT, and the increment was more evident in the treatment with CM from the U87-MG cells irradiated with mPDT (*p* < 0.01) (Figure 5). In this experiment, CM was obtained from the U87-MG cells exposed to CPN (6 mg/L) and irradiated with 10 J/cm^2^ in both modalities for comparison. PDT is associated with the modulation of immune and inflammatory responses as a one of the main antitumoral mechanisms. Some studies have shown the potentiality of PDT with different PSs to eliminate or reprogram TAM populations. For instance, Soyama T. et al. synthesized a mannose-conjugated chlorin e6 to decrease the proportion of M2-TAMs and increase that of M1-like TAMs [71]. A recent study of Lerouge L. et al. demonstrated that the secretome of post-PDT GBM cells treated with AGuIX^®^ nanoparticles polarized macrophages to an M1-like phenotype [68]. Our results were in agreement with this finding, and, in our case, the mPDT irradiation modality increased the overexpression of M1 marker transcripts significantly compared to the conventional modality.

**Figure 6:**
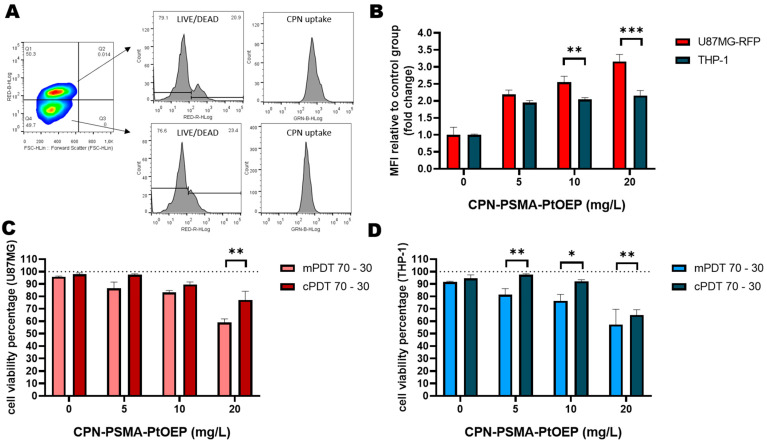
PDT evaluation in GBM/TAMs co-cultures. (**A**) Representative gate strategy by FC to differentiate GBM U87MG RFP+ cells from non-labeled macrophages and subsequent live/dead cell evaluation (ged-R channel) and CPN cell incorporation by fluorescence detection in the green-B channel. (**B**) Cell uptake quantification in U87-MGRFP cells and THP-1-derived macrophages from co-cultures exposed to increasing CPN concentrations and relative to mean fluorescence intensity from control groups without CPN incubation. (**C**) Cell viability percentages of U87-MGRFP cells from 2:1 co-culture ratio after 24 h post mPDT or cPDT treatment and evaluated by FC using LIVE/DEAD™ Fixable Far Red staining kit. (**D**) Cell viability percentages of THP-1-derived macrophages from 2:1 co-culture ratio after 24 h post mPDT or cPDT and evaluated by FC using LIVE/DEAD™ Fixable Far Red staining kit. * *p* < 0.05; ** *p* < 0.01, *** *p* < 0.001.

In the co-culture conditions, both cell types were evaluated in their differential CPN uptake ability and their response to PDT treatment. To accomplish this, the co-cultured GBM/TAMs were incubated with CPNs for 24 h according to the protocol proposed in monocultures of GBM cell lines (vide supra). Furthermore, PDT was carried out in both modalities at 10 J/cm^2^, and cell viability was evaluated by FC in the Q1 and Q4 quartile cell population of the FSC vs. RED-B channel dot plot graphs, which corresponded to tumor cells (Q1) and macrophages (Q4), respectively (Figure 6A). CPN intrinsic fluorescence from these same quartiles was used to compare the cellular uptake of nanoparticles. We observed that the particle uptake in both cell types was in a concentration-dependent manner by FC. The GBM cells were able to significantly incorporate more nanoparticles than the TAMs, even at the highest CPN concentrations of 10 and 20 mg/L (Figure 6B).

In the co-culture conditions, the data showed that the dark toxicity of CPNs was null in both cell types at the concentrations evaluated (Figure S6). However, a cytotoxic effect was observed in both cell types after the CPN-PDT treatment with the two modalities (Figure 6C,D). Nevertheless, the survival response of the U87-MG tumor cells was not similar to that in the monocultures of this cell line (vide supra). The cell viability of the U87-MG cells was greater in the co-culture conditions at a ratio of 2:1 (70,000 GBM cells/30,000 macrophages) in all the CPN concentrations evaluated compared to in the monoculture conditions (Figure 6C). The macrophages were also affected in their cell viability in a CPN-concentration-dependent manner (Figure 6D). We suggested that macrophages may exert a cytoprotective effect by competing for the cellular incorporation of nanoparticles. This phenomenon was even more noticeable in the culture conditions at a ratio of 1:1 (50,000 GBM cells/50,000 macrophages) (Figure S7). However, the mPDT modality continued to be more effective in eliminating the population of tumor cells as well as TAMs. It should be noted that the surface functionalization of the CPNs with specific ligands was not carried out and evaluated in the present study to selectively attack any of the populations inquired. However, strategies that aim to selectively eliminate a cell population with photo-assisted treatments have been previously described by other groups as well as us, demonstrating the potential of this type of more selective treatment [33,68].

**Figure 7:**
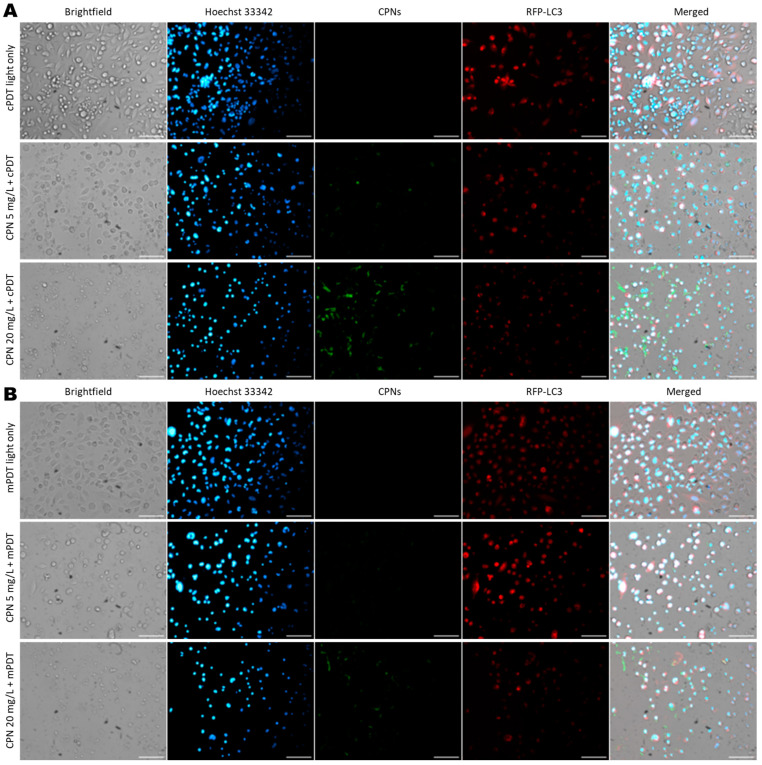
Cell morphologic changes in GBM/TAMs co-cultures after PDT. Representative fluorescence and brightfield images of co-cultures of U87-MGRFP+- and THP-1-derived macrophages exposed to increasing CPN concentrations for 24 h and irradiated with 10 J/cm2 in cPDT (**A**) or mPDT (**B**) modalities. Cells were stained with Hoechst 33342 at 1 µg/mL for 10 min for nuclei visualization, and RFP and CPN were also visualized in their respective channels. Scale bar = 100 µm.

The cell uptake by macrophages and GBM cells was confirmed by fluorescence microscopy (Figure 7). Based on cell type discrimination by the RFP+ GBM cells against non-staining macrophages, it was possible to visualize CPN fluorescence in both cell types, and the intracellular CPN fluorescence increased as a function of the nanoparticle concentration (Figure 7). The cell cytotoxicity of PDT in the two irradiation modalities was evident in the brightfield images of treated cells, which showed changes in the cell phenotype with an apoptotic appearance, a loss of cell membrane integrity, cell detachment and debris. These changes were more evident in the mPDT group compared to cPDT group; however, fewer changes in cell death were observed in the co-cultures compared to the monocultures of U87-MG tumor cells at equivalent light doses and CPN concentrations.

### 3.6. In Vivo Evaluation of PDT with CPNs in GBM Xenograft Mouse Model

**Figure 8:**
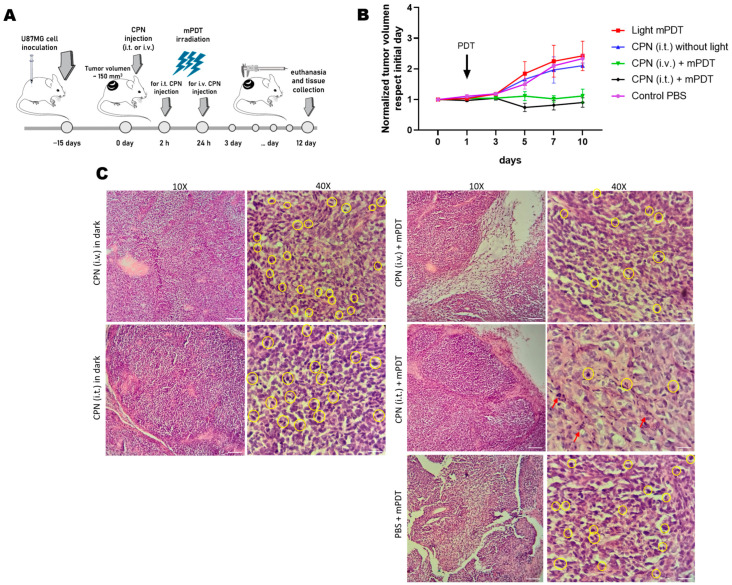
In vivo mPDT evaluation in GBM xenograft BALB/c nude mice model. (**A**) Schematic representation of protocol used for PDT assay. Mice were inoculated with U87-MG cells (2 × 10^6^ cells) in the flank. At day 15, when the tumors had an average size of ~150 mm^3^, CPN was injected intravenously (i.v.) or intratumorally (i.t.). PDT was performed by illumination of the tumor on the flank with 460 nm fiber-optic light at 12 mW/cm^2^ for 30 min. (**B**) Tumor growth curves normalized to initial volume tumors of mice of different treatment conditions; the tumor volume was measured with a caliper every 2 days up to 10 days after PDT irradiation. (**C**) Representative histological images (40× and 10×) of tumor tissues at day-12 time point after PDT treatment. Yellow circles represent mitotic figures in the different treatments, and red arrows mark apoptotic events. The scale bars represent 200 µm (10×) and 50 µm (40×), respectively.

To explore the potential of CPN using mPDT irradiation protocols in animal models, we developed U87-MG xenograft tumors in BALB/c nude mice and performed mPDT irradiation for 30 min. with a system of a fiber optic coupled with a multi-LED (Figure S8) after either i.v. or i.t. administration of CPN (Figure 8A).

The mice treated with a single i.t. or i.v. administration of CPNs displayed a strongly inhibited tumor growth for 10 days after PDT treatment (Figure 8B). Tumors from the PBS control or CPN non-irradiated groups reached more than twice their initial volume. Moreover, we did not observe changes in mice weight, which indicated that there was no treatment-induced toxicity. These findings were also supported in the histological evaluation of organ tissues such as the spleen, liver and kidneys where no morphological changes were evident in any of the treated groups or in the control group with PBS (Figure S9).

The inhibitory tumor growth was superior in the i.t. administration compared to the i.v. administration of CPNs, and this could be attributed to the immediate nanoparticle intratumoral accumulation achieved with this modality [54]. Even though the i.t. administration resulted in a higher and more constant accumulation overtime, it is important to consider that i.v. administration is a more realistic administration route, as most of the tumors in humans are not visible as is the case in subcutaneous animal models of cancer. In the latter, the intratumoral accumulation of CPN was achieved probably due to the enhanced permeability and retention (EPR) effect [54]. Despite the fact that heterotopically developed GBM tumors do not reproduce blood–brain barrier characteristics, our intention was to evaluate the in vivo efficacy of PDT for the first time with this type of CPN, which has shown great promise in previous in vitro assays [1,16]. After the euthanasia of the animals, the histological analysis of the tumor tissues corresponding to the different treatment groups was performed. In the samples corresponding to the group treated with PBS and irradiated with the mPDT modality, an infiltrative tumor proliferation was observed with an irregular disposition of the neoplastic tissue and the presence of blood vessels of a medium caliber. At a higher magnification, pleomorphism and an intense mitotic index (yellow circles) were observed (Figure 8C). These characteristics were also evident in samples from the groups treated with CPN (i.t. or i.v.) without irradiation exposure. It is noteworthy that the mitotic index continued to be high with typical and atypical mitotic figures and corresponded to the sustained growth of tumors in these groups. The formation of small capillary blood vessels within the tumors was also observed. On the other hand, the tumors treated with CPNs and irradiated displayed circumscribed tumor tissues with lax isolated areas of a myxoid appearance and with the development of connective tissue that infiltrated and where tumor proliferation was not as compact. On the surface, the presence of mononuclear inflammatory cells was observed. These findings were more marked in the tumors treated with CPN administered i.t. and irradiated and where the presence of many apoptotic bodies was also evidenced (Figure 8C). These findings add to those reported with other types of molecular PSs activated with blue light in PDT in vivo protocols [72,73]. Future experiments are being planned to evaluate PDT with CPNs using a low light fluence rate in an orthotopic model.

The intensity of the light used in PDT or the fluence rate is an important factor to consider for the design of optimal PDT protocols [66], Henderson B. et al. demonstrated that severe oxygen depletion is shown to occur within seconds of illumination at a fluence rate of 75 mW/cm^2^ in preclinical tumor mice models in vivo, and a fluence rate of 14 mW/cm^2^ was found to be optimal for tumor elimination in their setting [74]. These data show that higher fluence rates are not necessarily more efficient in inducing photodynamic effects and cytotoxicity. Clinical studies have demonstrated a benefit for patients treated with molecular-PS-mediated PDT in terms of the prolongation of median overall survival (OS) as well as quality of life [8]. A recent study evaluated PDT clinically for malignant brain tumors in children and young adolescents using talaporfin sodium with a moderate benefit in terms of overall survival and progression-free survival [75]. However, the disadvantage of PDT is the depth of the light penetration; therefore, maximum surgical resection is mandatory to achieve optimal effects. An alternative irradiation modality is the interstitial PDT delivery of light, which offers improvement outcomes in terms of OS using 5-ALA as molecular PS [76]. From a clinical perspective, it is expected that more third-generation PSs such as those assayed in this study will reach clinical trials, and with suitable light doses regimes it is possible to overcome the obstacles presented by PDT, such as the abrupt consumption of oxygen and the activation of molecular pathways of therapeutic resistance.

## 4. Conclusions

In this work, we reported on the evaluation of CPN for the PDT of GBM tumors. We assayed these nanoparticles in different irradiation modalities with a low and high light fluence rates to exploit the potential of CPNs to achieve the photokilling of GBM cell lines, thus dropping the possible mechanisms of therapeutic resistance developed as a tumor adaptation. Using monocultures and co-cultures in in vitro models simulating a tumor microenvironment, we observed that CPNs were preferentially taken up by GBM cells when compared to TAMs derived from THP-1 cells in co-culture conditions. After light exposure in a regular GBM culture model, mPDT promoted total tumor cell death in very low CPN concentrations, but TAMs exerted a cytoprotective action in the co-culture models. The mPDT irradiation modality was able to trigger significant photokilling in all the GBM cell lines through different cell death mechanisms compared to the conventional modality. We determined that CPNs in mPDT irradiation protocols slightly promoted the apoptosis of GBM cells. In addition, the mPDT modality polarized TAMs to a proinflammatory profile that would probably contribute to an overall therapeutic improvement. In a GBM animal model, CPNs in mPDT in vivo protocols were able to significantly slow tumor growth and also generated cell death with evident histological changes. Overall, these data provide valuable information about the development of optimized PDT protocols with lower light irradiance and CNPs for GBM management.

## Figures and Tables

**Table 1:** qPCR primers designed on Primer-BLAST and verified on BLAST-N (NCBI).

Marker	Gene	Forward 5′-3′	Reverse 3′-5′	Length (bp)	NM
M1	TNFα	GAGGCCAAGCCCTGGTATG	CGGGCCGATTGATCTCAGC	91	NM_000594.4
	CCR7	TGAGGTCACGGACGATTACAT	GTAGGCCCACGAAACAAATGAT	143	NM_001838.4
M2	MRC-1	GGGTTGCTATCACTCTCTATGC	TTTCTTGTCTGTTGCCGTAGTT	126	NM_002438.4
	VEGFA	GGCGAGGCAGCTTGAGTTAA	CACCGCCTCGGCTTGTC	57	NM_001025366.3
Ref.	GADPH	GACCTGACCTGCCGTCTAGAAAAA	ACCACCCTGTTGCTGTAGCCAAAT	245	NM_001357943.2

**Table 2:** Average IC_50_ CPN concentration (mg/L) for both PDT irradiation modalities in GBM cell lines.

IC_50_ *	U87-MG	T98G	M059K
mPDT	0.8 (40 J/cm^2^)	24.7 (40 J/cm^2^)	1.2 (40 J/cm^2^)
	4.5 (10 J/cm^2^)	31.7 (10 J/cm^2^)	9 (10 J/cm^2^)
cPDT	3.5 (40 J/cm^2^)	27.6 (40 J/cm^2^)	4.7 (40 J/cm^2^)
	11.3 (10 J/cm^2^)	39.6 (10 J/cm^2^)	17.4 (10 J/cm^2^)

* Values obtained by non-linear regression fitting of MTT viability assays in GraphPad software (version 8.2.1).

## Data Availability

All other data are available from the corresponding author upon reasonable request.

## Supplementary Materials

The following supporting information can be downloaded at: https://www.mdpi.com/article/10.3390/cells12111541/s1, Figure S1. CPN spectra and size characterization; Figure S2. Cell viability assay by MTT for MO59K; Figure S3. CPN cell uptake evaluation in GBM cell lines with FC; Figure S4. Co-cultures of U87-MGRFP and THP-1-derived macrophages; Figure S5. Gene expression of macrophage M1 and M2 phenotype evaluation after CM exposure of GBM cells; Figure S6. Biocompatibility evaluation of CPN-PSMA-PtOEP in co-cultures of GBM/TAMs; Figure S7. PDT evaluation in a GBM/TAMs co-culture 1:1 ratio; Figure S8. GBM xenograft mouse model and mPDT in vivo protocol. Figure S9. Histological evaluation of detoxifying organs after mPDT protocol.

## References

[1] Ibarra L.E., Porcal G.V., Macor L.P., Ponzio R.A., Spada R.M., Lorente C., Chesta C.A.C.A., Rivarola V.A., Palacios R.E. (2018). Metallated porphyrin-doped conjugated polymer nanoparticles for efficient photodynamic therapy of brain and colorectal tumor cells. Nanomedicine.

[2] Ibarra L.E., Beaugé L., Arias-Ramos N., Rivarola V.A., Chesta C.A., López-Larrubia P., Palacios R.E. (2020). Trojan horse monocyte-mediated delivery of conjugated polymer nanoparticles for improved photodynamic therapy of glioblastoma. Nanomedicine.

[3] Ibarra L.E., Vilchez M.L., Caverzán M.D., Milla Sanabria L.N. (2021). Understanding the glioblastoma tumor biology to optimize photodynamic therapy: From molecular to cellular events. J. Neurosci. Res..

[4] Bartusik-Aebisher D., Żołyniak A., Barnaś E., Machorowska-Pieniążek A., Oleś P., Kawczyk-Krupka A., Aebisher D. (2022). The Use of Photodynamic Therapy in the Treatment of Brain Tumors—A Review of the Literature. Molecules.

[5] Akimoto J. (2016). Photodynamic therapy for malignant brain tumors. Neurol. Med. Chir..

[6] Leroy H.A., Baert G., Guerin L., Delhem N., Mordon S., Reyns N., Vignion-Dewalle A.S. (2021). Interstitial Photodynamic Therapy for Glioblastomas: A Standardized Procedure for Clinical Use. Cancers.

[7] Kostron H., Obwegeser A., Jakober R. (1996). Photodynamic therapy in neurosurgery: A review. J. Photochem. Photobiol. B Biol..

[8] Kostron H. (2010). Photodynamic diagnosis and therapy and the brain. Methods Mol. Biol..

[9] Foresto E., Gilardi P., Ibarra L.E., Cogno I.S. (2021). Light-activated green drugs: How we can use them in photodynamic therapy and mass-produce them with biotechnological tools. Phytomed. Plus.

[10] Spada R.M., Macor L.P., Hernández L.I., Ponzio R.A., Ibarra L.E., Lorente C., Chesta C.A., Palacios R.E. (2018). Amplified singlet oxygen generation in metallated-porphyrin doped conjugated polymer nanoparticles. Dyes Pigment..

[11] Bacellar I.O.L., Tsubone T.M., Pavani C., Baptista M.S. (2015). Photodynamic efficiency: From molecular photochemistry to cell death. Int. J. Mol. Sci..

[12] Kuimova M.K., Yahioglu G., Ogilby P.R. (2009). Singlet oxygen in a cell: Spatially dependent lifetimes and quenching rate constants. J. Am. Chem. Soc..

[13] Schmitt F.J., Renger G., Friedrich T., Kreslavski V.D., Zharmukhamedov S.K., Los D.A., Kuznetsov V.V., Allakhverdiev S.I. (2014). Reactive oxygen species: Re-evaluation of generation, monitoring and role in stress-signaling in phototrophic organisms. Biochim. Biophys. Acta-Bioenerg..

[14] Mahmoudi K., Garvey K.L., Bouras A., Cramer G., Stepp H., Jesu Raj J.G., Bozec D., Busch T.M., Hadjipanayis C.G. (2019). 5-aminolevulinic acid photodynamic therapy for the treatment of high-grade gliomas. J. Neurooncol..

[15] Kim M.M., Darafsheh A. (2020). Light Sources and Dosimetry Techniques for Photodynamic Therapy. Photochem. Photobiol..

[16] Caverzán M.D., Beaugé L., Chesta C.A., Palacios R.E., Ibarra L.E. (2020). Photodynamic therapy of Glioblastoma cells using doped conjugated polymer nanoparticles: An in vitro comparative study based on redox status. J. Photochem. Photobiol. B Biol..

[17] Hambardzumyan D., Bergers G. (2015). Glioblastoma: Defining Tumor Niches. Trends Cancer.

[18] Chédeville A.L., Lourdusamy A., Monteiro A.R., Hill R., Madureira P.A. (2020). Investigating glioblastoma response to hypoxia. Biomedicines.

[19] Algorri J.F., Ochoa M., Roldán-Varona P., Rodríguez-Cobo L., López-Higuera J.M. (2021). Light technology for efficient and effective photodynamic therapy: A critical review. Cancers.

[20] Kirino I., Fujita K., Sakanoue K., Sugita R., Yamagishi K., Takeoka S., Fujie T., Uemoto S., Morimoto Y. (2020). Metronomic photodynamic therapy using an implantable LED device and orally administered 5-aminolevulinic acid. Sci. Rep..

[21] Rego-Filho F.G., De Araujo M.T., De Oliveira K.T., Bagnato V.S. (2014). Validation of photodynamic action via photobleaching of a new curcumin-based composite with enhanced water solubility. J. Fluoresc..

[22] James N.S., Cheruku R.R., Missert J.R., Sunar U., Pandey R.K. (2018). Measurement of cyanine dye photobleaching in photosensitizer cyanine dye conjugates could help in optimizing light dosimetry for improved photodynamic therapy of cancer. Molecules.

[23] Lamberti M.J., Pansa M.F., Vera R.E., Fernández-Zapico M.E., Vittar N.B.R., Rivarola V.A. (2017). Transcriptional activation of HIF-1 by a ROSERK axis underlies the resistance to photodynamic therapy. PLoS ONE.

[24] Zhan Q., Yue W., Hu S. (2011). Effect of photodynamic therapy and endostatin on human glioma xenografts in nude mice. Photodiagn. Photodyn. Ther..

[25] Milla Sanabria L., Rodríguez M.E., Cogno I.S., Rumie Vittar N.B., Pansa M.F., Lamberti M.J., Rivarola V.A. (2013). Direct and indirect photodynamic therapy effects on the cellular and molecular components of the tumor microenvironment. Biochim. Biophys. Acta-Rev. Cancer.

[26] Simsek C., Esin E., Yalcin S. (2019). Metronomic Chemotherapy: A Systematic Review of the Literature and Clinical Experience. J. Oncol..

[27] Zhou Q., Guo P., Wang X., Nuthalapati S., Gallo J.M. (2007). Preclinical pharmacokinetic and pharmacodynamic evaluation of metronomic and conventional temozolomide dosing regimens. J. Pharmacol. Exp. Ther..

[28] Yamagishi K., Kirino I., Takahashi I., Amano H., Takeoka S., Morimoto Y., Fujie T. (2018). Tissue-adhesive wirelessly powered optoelectronic device for metronomic photodynamic cancer therapy. Nat. Biomed. Eng..

[29] Bogaards A., Varma A., Zhang K., Zach D., Bisland S.K., Moriyama E.H., Lilge L., Muller P.J., Wilson B.C. (2005). Fluorescence image-guided brain tumour resection with adjuvant metronomic photodynamic therapy: Pre-clinical model and technology development. Photochem. Photobiol. Sci..

[30] Bisland S.K., Lilge L., Lin A., Rusnov R., Wilson B.C. (2004). Metronomic Photodynamic Therapy as a New Paradigm for Photodynamic Therapy: Rationale and Preclinical Evaluation of Technical Feasibility for Treating Malignant Brain Tumors. Photochem. Photobiol..

[31] Zhang C., Yuan Q., Zhang Z., Tang Y. (2023). A pH-Responsive Drug Delivery System Based on Conjugated Polymer for Effective Synergistic Chemo-/Photodynamic Therapy. Molecules.

[32] Pham T.T.D., Jung S.J., Oh C.M., Yang J.K., Lee D., Kidanemariam A., Muhammad A., Kim S., Shin T.J., Park J.H. (2023). Conjugated Polymer Nanoparticles: Photothermal and Photodynamic Capabilities According to Molecular Ordering in Their Assembly Structures. Macromolecules.

[33] Ibarra L.E., Camorani S., Agnello L., Pedone E., Pirone L., Chesta C.A., Palacios R.E., Fedele M., Cerchia L. (2022). Selective Photo-Assisted Eradication of Triple-Negative Breast Cancer Cells through Aptamer Decoration of Doped Conjugated Polymer Nanoparticles. Pharmaceutics.

[34] Ponzio R.A., Ibarra L.E., Achilli E.E., Odella E., Chesta C.A., Martínez S.R., Palacios R.E. (2022). Sweet light o’ mine: Photothermal and photodynamic inactivation of tenacious pathogens using conjugated polymers. J. Photochem. Photobiol. B Biol..

[35] Ibarra L.E. (2022). Development of nanosystems for active tumor targeting in photodynamic therapy. Ther. Deliv..

[36] Chen Z., Feng X., Herting C.J., Garcia V.A., Nie K., Pong W.W., Rasmussen R., Dwivedi B., Seby S., Wolf S.A. (2017). Cellular and molecular identity of tumor-associated macrophages in glioblastoma. Cancer Res..

[37] Caverzán M.D., Beaugé L., Oliveda P.M., González B.C., Bühler E.M., Ibarra L.E. (2023). Exploring Monocytes-Macrophages in Immune Microenvironment of Glioblastoma for the Design of Novel Therapeutic Strategies. Brain Sci..

[38] Costa E.C., Gaspar V.M., Marques J.G., Coutinho P., Correia I.J. (2013). Evaluation of Nanoparticle Uptake in Co-culture Cancer Models. PLoS ONE.

[39] Foglietta F., Pinnelli V., Giuntini F., Barbero N., Panzanelli P., Durando G., Terreno E., Serpe L., Canaparo R. (2021). Sonodynamic Treatment Induces Selective Killing of Cancer Cells in an In Vitro Co-Culture Model. Cancers.

[40] Gallastegui A., Spada R.M., Cagnetta G., Ponzio R.A., Martínez S.R., Previtali C.M., Gómez M.L., Palacios R.E., Chesta C.A. (2020). Conjugated Polymer Nanoparticles as Unique Coinitiator-Free, Water-Soluble, Visible-Light Photoinitiators of Vinyl Polymerization. Macromol. Rapid Commun..

[41] Lamberti M.J., Morales Vasconsuelo A.B., Ferrara M.G., Rumie Vittar N.B. (2020). Recapitulation of Hypoxic Tumor–stroma Microenvironment to Study Photodynamic Therapy Implications. Photochem. Photobiol..

[42] Starr T., Bauler T.J., Malik-Kale P., Steele-Mortimer O. (2018). The phorbol 12-myristate-13-acetate differentiation protocol is critical to the interaction of THP-1 macrophages with Salmonella Typhimurium. PLoS ONE.

[43] Herold-Mende C., Linder B., Andersen J.K., Miletic H., Hossain J.A. (2022). Tumor-Associated Macrophages in Gliomas&mdash;Basic Insights and Treatment Opportunities. Cancers.

[44] Georgieva P.B., Mathivet T., Alt S., Giese W., Riva M., Balcer M., Gerhardt H. (2020). Long-lived tumor-associated macrophages in glioma. Neuro-Oncol. Adv..

[45] Chen Z., Ross J.L., Hambardzumyan D. (2019). Intravital 2-photon imaging reveals distinct morphology and infiltrative properties of glioblastoma-associated macrophages. Proc. Natl. Acad. Sci. USA.

[46] Lu-Emerson C., Snuderl M., Kirkpatrick N.D., Goveia J., Davidson C., Huang Y., Riedemann L., Taylor J., Ivy P., Duda D.G. (2013). Increase in tumor-associated macrophages after antiangiogenic therapy is associated with poor survival among patients with recurrent glioblastoma. Neuro Oncol..

[47] Chen Z., Hambardzumyan D. (2021). Macrophage-tumor cell intertwine drives the transition into a mesenchymal-like cellular state of glioblastoma. Cancer Cell.

[48] Martínez S.R., Ibarra L.E., Ponzio R.A., Forcone M.V., Wendel A.B., Chesta C.A., Spesia M.B., Palacios R.E. (2020). Photodynamic Inactivation of ESKAPE Group Bacterial Pathogens in Planktonic and Biofilm Cultures Using Metallated Porphyrin-Doped Conjugated Polymer Nanoparticles. ACS Infect. Dis..

[49] Eruslanov E., Kusmartsev S. (2010). Identification of ROS using oxidized DCFDA and flow-cytometry. Methods Mol. Biol..

[50] Soriano Pérez M.L., Funes J.A., Flores Bracamonte C., Ibarra L.E., Forrellad M.A., Taboga O., Cariddi L.N., Salinas F.J., Ortega H.H., Alustiza F. (2023). Development and biological evaluation of pNIPAM-based nanogels as vaccine carriers. Int. J. Pharm..

[51] Genin M., Clement F., Fattaccioli A., Raes M., Michiels C. (2015). M1 and M2 macrophages derived from THP-1 cells differentially modulate the response of cancer cells to etoposide. BMC Cancer.

[52] Maeß M.B., Sendelbach S., Lorkowski S. (2010). Selection of reliable reference genes during THP-1 monocyte differentiation into macrophages. BMC Mol. Biol..

[53] Daigneault M., Preston J.A., Marriott H.M., Whyte M.K.B., Dockrell D.H. (2010). The identification of markers of macrophage differentiation in PMA-stimulated THP-1 cells and monocyte-derived macrophages. PLoS ONE.

[54] Arias-Ramos N., Ibarra L.E., Serrano-Torres M., Yagüe B., Caverzán M.D., Chesta C.A., Palacios R.E., López-Larrubia P. (2021). Iron Oxide Incorporated Conjugated Polymer Nanoparticles for Simultaneous Use in Magnetic Resonance and Fluorescent Imaging of Brain Tumors. Pharmaceutics.

[55] Kersemans V., Cornelissen B., Allen P.D., Beech J.S., Smart S.C. (2013). Subcutaneous tumor volume measurement in the awake, manually restrained mouse using MRI. J. Magn. Reson. Imaging.

[56] Zhang Y., Dube C., Gibert M., Cruickshanks N., Wang B., Coughlan M., Yang Y., Setiady I., Deveau C., Saoud K. (2018). The p53 Pathway in Glioblastoma. Cancers.

[57] Maria Forte I., Indovina P., Antonella Iannuzzi C., Cirillo D., Marzo D.D.I., Barone D., Capone F., Pentimalli F., Giordano A. (2019). Targeted therapy based on p53 reactivation reduces both glioblastoma cell growth and resistance to temozolomide. Int. J. Oncol..

[58] Lee S.Y. (2016). Temozolomide resistance in glioblastoma multiforme. Genes Dis..

[59] Aguilar Cosme J.R., Gagui D.C., Green N.H., Bryant H.E., Claeyssens F. (2021). In Vitro Low-Fluence Photodynamic Therapy Parameter Screening Using 3D Tumor Spheroids Shows that Fractionated Light Treatments Enhance Phototoxicity. ACS Biomater. Sci. Eng..

[60] Busch T.M. (2006). Local physiological changes during photodynamic therapy. Lasers Surg. Med..

[61] Shi X., Zhang H., Jin W., Liu W., Yin H., Li Y., Dong H. (2019). Metronomic photodynamic therapy with 5-aminolevulinic acid induces apoptosis and autophagy in human SW837 colorectal cancer cells. J. Photochem. Photobiol. B Biol..

[62] Moserova I., Kralova J. (2012). Role of ER Stress Response in Photodynamic Therapy: ROS Generated in Different Subcellular Compartments Trigger Diverse Cell Death Pathways. PLoS ONE.

[63] Lange C., Lehmann C., Mahler M., Bednarski P.J. (2019). Comparison of Cellular Death Pathways after mTHPC-mediated Photodynamic Therapy (PDT) in Five Human Cancer Cell Lines. Cancers.

[64] Fernando L.P., Kandel P.K., Yu J.B., McNeill J., Ackroyd P.C., Christensen K. (2010). a Mechanism of Cellular Uptake of Highly Fluorescent Conjugated Polymer Nanoparticles. Biomacromolecules.

[65] Villalpando-Rodriguez G.E., Gibson S.B. (2021). Reactive Oxygen Species (ROS) Regulates Different Types of Cell Death by Acting as a Rheostat. Oxid. Med. Cell. Longev..

[66] Huis in ‘t Veld R.V., Heuts J., Ma S., Cruz L.J., Ossendorp F.A., Jager M.J. (2023). Current Challenges and Opportunities of Photodynamic Therapy against Cancer. Pharmaceutics.

[67] Davies N., Wilson B.C. (2007). Interstitial in vivo ALA-PpIX mediated metronomic photodynamic therapy (mPDT) using the CNS-1 astrocytoma with bioluminescence monitoring. Photodiagn. Photodyn. Ther..

[68] Lerouge L., Gries M., Chateau A., Daouk J., Lux F., Rocchi P., Cedervall J., Olsson A.-K., Tillement O., Frochot C. (2023). Targeting Glioblastoma-Associated Macrophages for Photodynamic Therapy Using AGuIX®-Design Nanoparticles. Pharmaceutics.

[69] Gattas M.J., Estecho I.G., Lago Huvelle M.A., Errasti A.E., Carrera Silva E.A., Simian M. (2021). A heterotypic tridimensional model to study the interaction of macrophages and glioblastoma in vitro. Int. J. Mol. Sci..

[70] Park J.V., Chandra R., Cai L., Ganguly D., Li H., Toombs J.E., Girard L., Brekken R.A., Minna J.D. (2022). Tumor Cells Modulate Macrophage Phenotype in a Novel In Vitro Co-Culture Model of the NSCLC Tumor Microenvironment. J. Thorac. Oncol..

[71] Soyama T., Sakuragi A., Oishi D., Kimura Y., Aoki H., Nomoto A., Yano S., Nishie H., Kataoka H., Aoyama M. (2021). Photodynamic therapy exploiting the anti-tumor activity of mannose-conjugated chlorin e6 reduced M2-like tumor-associated macrophages. Transl. Oncol..

[72] Akasov R.A., Sholina N.V., Khochenkov D.A., Alova A.V., Gorelkin P.V., Erofeev A.S., Generalova A.N., Khaydukov E.V. (2019). Photodynamic therapy of melanoma by blue-light photoactivation of flavin mononucleotide. Sci. Rep..

[73] Mugas M.L., Calvo G., Marioni J., Céspedes M., Martinez F., Vanzulli S., Sáenz D., Di Venosa G., Nuñez Montoya S., Casas A. (2021). Photosensitization of a subcutaneous tumour by the natural anthraquinone parietin and blue light. Sci. Rep..

[74] Henderson B.W., Busch T.M., Snyder J.W. (2006). Fluence rate as a modulator of PDT mechanisms. Lasers Surg. Med..

[75] Chiba K., Aihara Y., Oda Y., Fukui A., Tsuduki S., Saito T., Nitta M., Muragaki Y., Kawamata T. (2022). Photodynamic therapy for malignant brain tumors in children and young adolescents. Front. Oncol..

[76] Foglar M., Aumiller M., Bochmann K., Buchner A., El Fahim M., Quach S., Sroka R., Stepp H., Thon N., Forbrig R. (2023). Interstitial Photodynamic Therapy of Glioblastomas: A Long-Term Follow-up Analysis of Survival and Volumetric MRI Data. Cancers.

