# Supplementary material for: Metronomic Photodynamic Therapy with Conjugated Polymer Nanoparticles in Glioblastoma Tumor Microenvironment
Source: Cells. 2023 Jun 4;12(11):1541. doi: 10.3390/cells12111541 (PMC10252555; doi:10.3390/cells12111541)
Supplement: Supplementary file 1 [file cells-12-01541-s001.zip › cells-2399146-supplementary.pdf]

Supplementary Data

Metronomic photodynamic therapy with conjugated polymer nanoparticles in glioblastoma tumor microenvironment

Supplementary Results

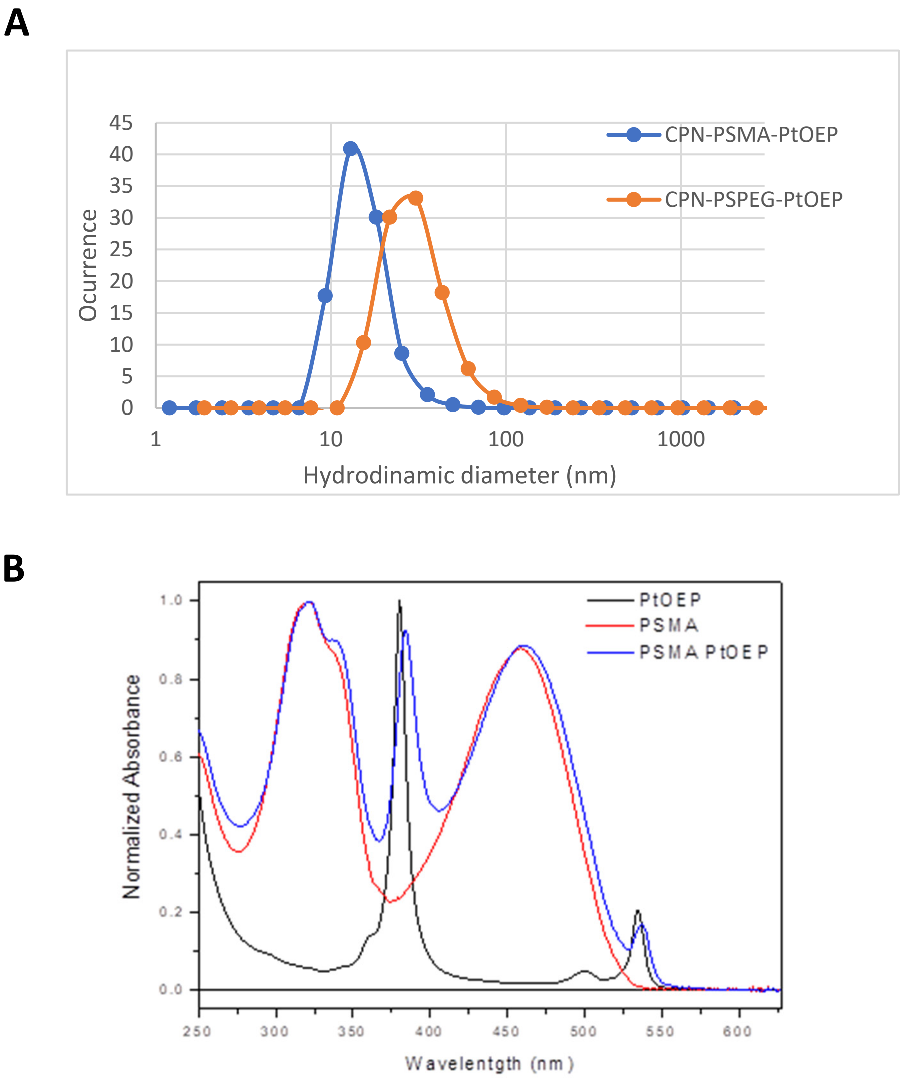

Figure S1. CPN size and spectra characterization. A) Hydrodynamic diameter histogram obtained by DLS of CPN-PSMA-PtOEP and CPN-PSPEG-PtOEP suspended in water. B) Spectra characterization of CPNs stabilized with PSMA in water, PtOEP in deoxygenated THF and CPNs-PSMA-PtOEP in water.

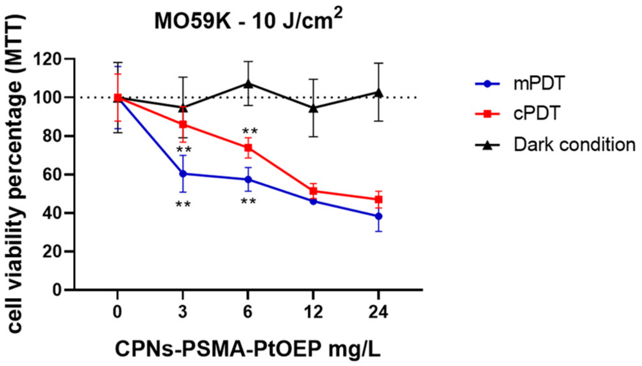

Figure S2. Cell viability assay by MTT for MO59K. Citotoxicity of different CPN concentration during incubation and 10 J/cm^2 light dose using both irradiance fluence density corresponding to mPDT (blue lines) and cPDT (red lines). Incubation with CPNs in dark is represented by black lines. Cell viability percentages were normalized to control cells exposed to light irradiation only. ** $p < 0.01$ with ANOVA test.

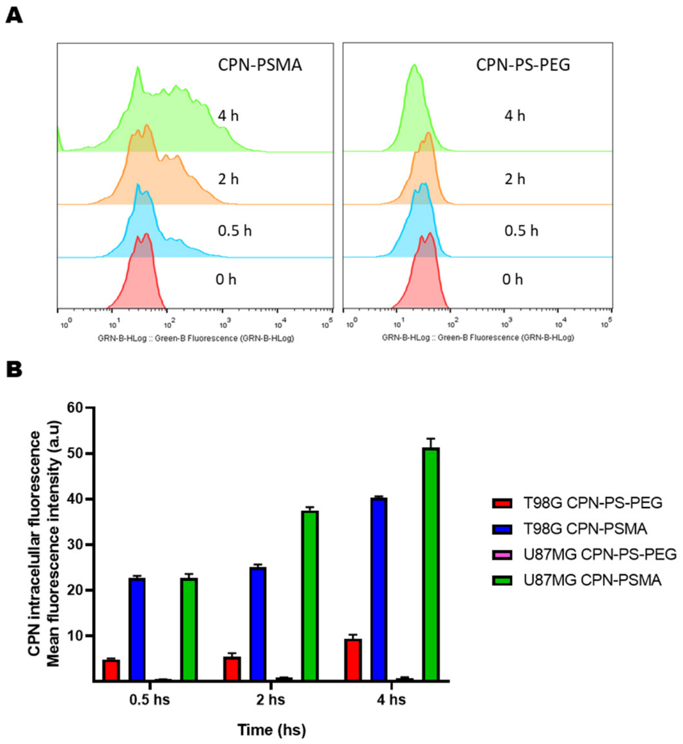

Figure S3. CPN cell uptake evaluation in GBM cell lines with FC. A) Green-fluorescence histograms from T98G cells exposed to 6 mg/L CPN-PSMA for different time periods (0, 0.5, 2 and 4 hours). B) CPN cellular uptake as a function of incubation. Bar graph represents the geometric mean fluorescence intensity in the green channel of cell populations of two independent experiments, and 0 h represents autofluorescence value from the negative control group (not exposed to CPNs).

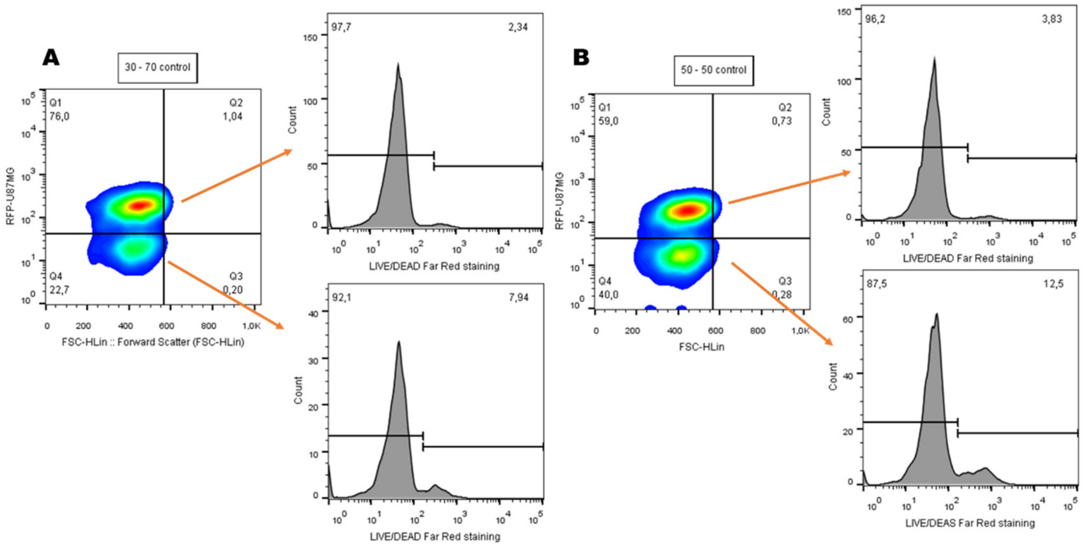

Figure S4. Co-cultures of U87-MGRFP and THP-1-derived macrophages. A) Flow cytometry analysis of co-cultures of RFP + GBM cells (70 % proportion) and non-staining macrophages (30% proportion) in a dot-plot graph (left panel) and their corresponding cell viability of both cell types evaluated by LIVE/DEAD far red staining kit (right panels). B) Flow cytometry analysis of co-cultures of RFP + GBM cells (50 % proportion) and non-staining macrophages (50% proportion) in a dot-plot graph (left panel) and their corresponding cell viability of both cell types evaluated by LIVE/DEAD far red staining kit (right panels). Cell viability was superior to 90% in both cell types under conventional culture conditions.

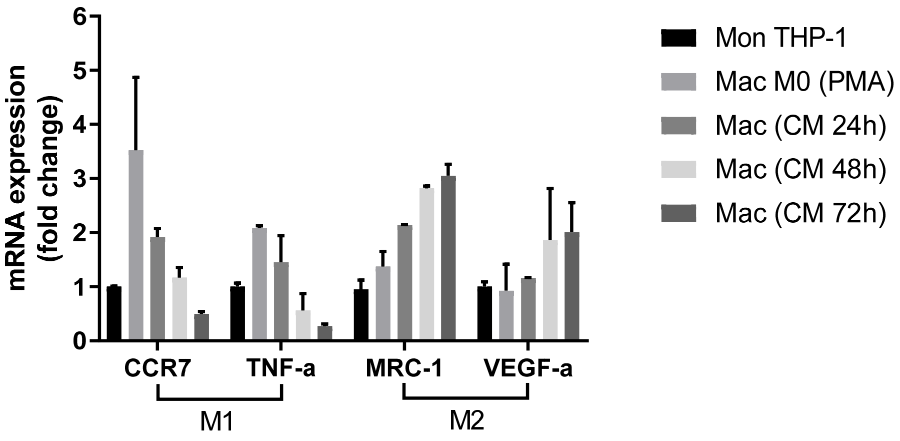

Figure S5. Gene expression of macrophage M1 and M2 phenotypes evaluation after CM exposure of GBM cells. Mean relative gene expression of markers for M1 (CCR7 and TNF-a) and M2 (MRC-1 and VEGF-a) (n = 3 for each gene marker, mean ± SD) normalized to GADPH housekeeping and relative to THP-1-derived macrophages after PMA differentiation protocol and resting period of 24 h in complete growth medium or exposed to CM obtained from U87MG cells for different time periods.

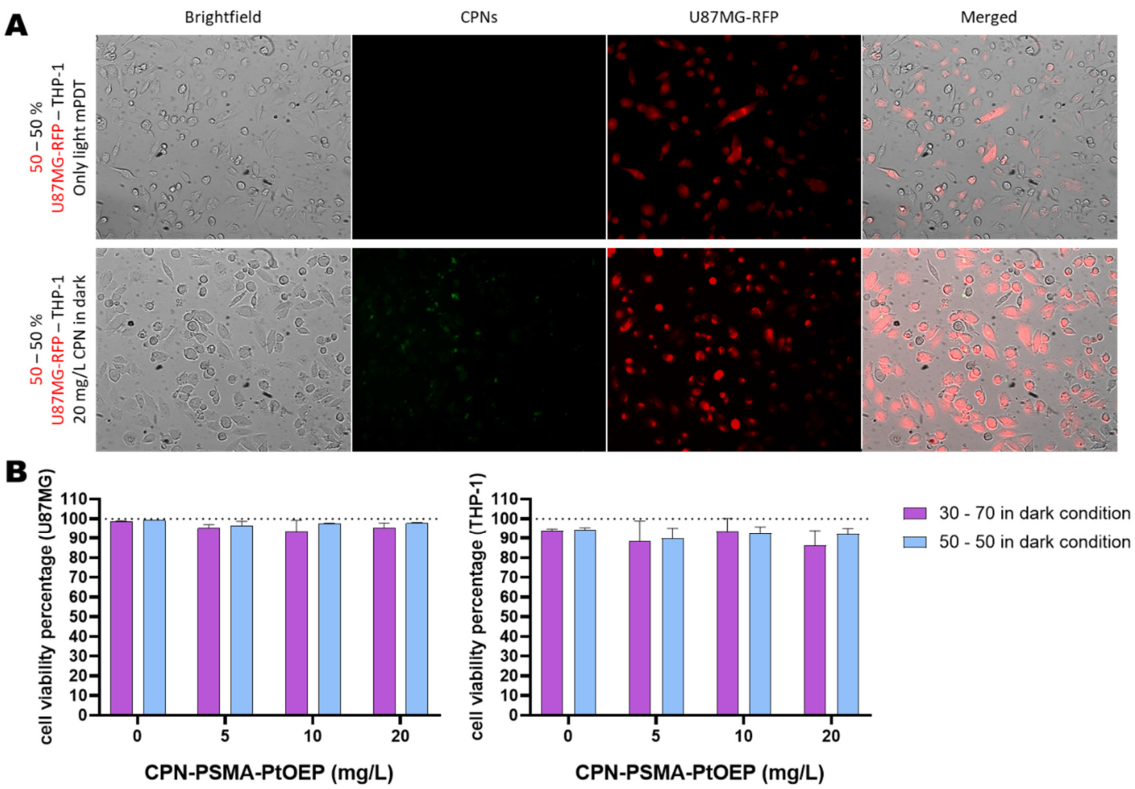

Figure S6. Biocompatibility evaluation of CPN-PSMA-PtOEP in co-cultures of GBM/TAMs. A) Representative fluorescence microscopy images of co-cultures of U87-MGRFP cells and THP-1-derived macrophages exposed to 10 mg/L of CPN-PSMA-PtOEP for 24 h without irradiation. B) Cell viability percentages in GBM U87MG and macrophages from co-cultures exposed to increasing CPN-PSMA-PtOEP concentrations for 24 h and evaluated by FC using LIVE/DEAD Far red staining kit.

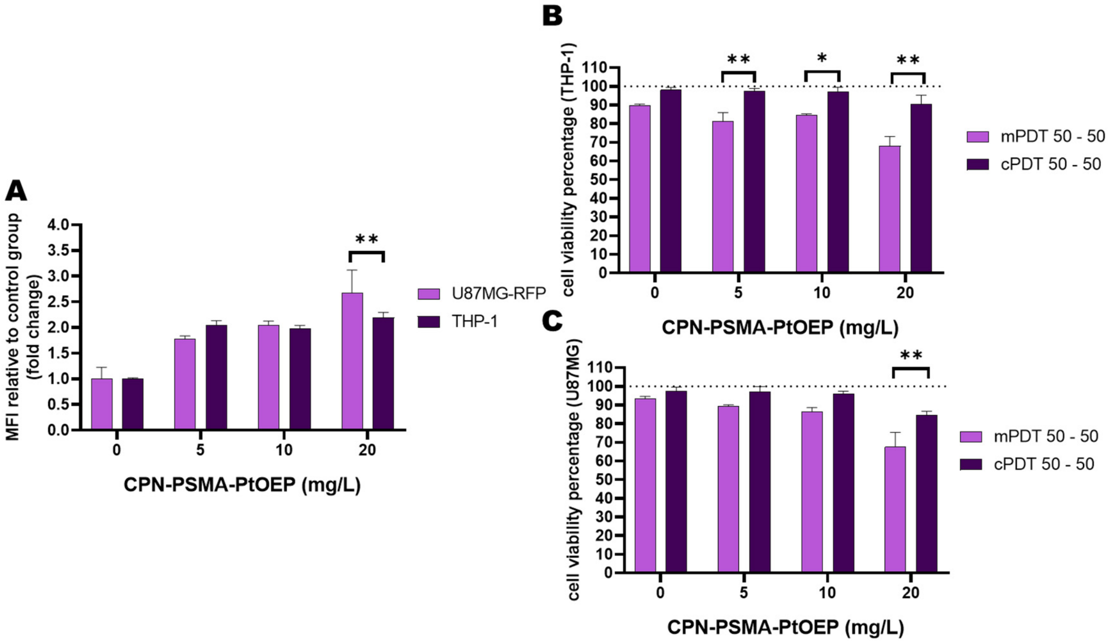

Figure S7. PDT evaluation in GBM/TAMs co-cultures 1:1 ratio. A) Cell uptake quantification in U87-MGRFP cells and THP-1-derived macrophages from co-cultures exposed to increasing CPN concentrations and relative to mean fluorescence intensity from control groups without CPN incubation. B) Cell viability

percentages of U87-MGRFP cells from 1:1 co-culture ratio after 24h post mPDT or cPDT and evaluated by FC using LIVE/DEAD™ Fixable Far Red staining kit. C) Cell viability percentages of THP-1-derived macrophages from 1:1 co-culture ratio after 24h post mPDT or cPDT and evaluated by FC using LIVE/DEAD™ Fixable Far Red staining kit

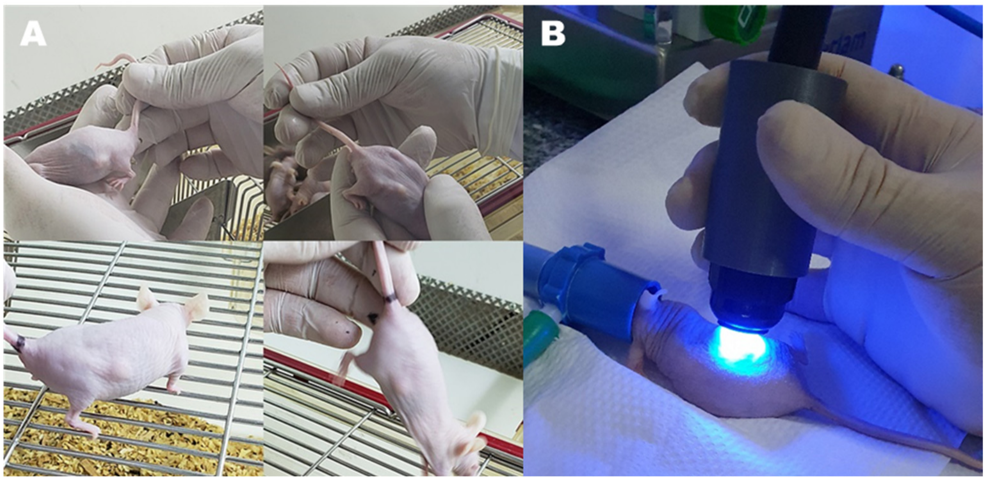

Figure S8. GBM xenograft mouse model and mPDT *in vivo* protocol. A) BALB/c nude mice were injected with 2×10^6 cells/0.1 ml/mouse and after approximately 15 days when tumors reach 150 mm^3, the protocol for the administration of nanoparticles and irradiation with fiber optics was carried out. B) .

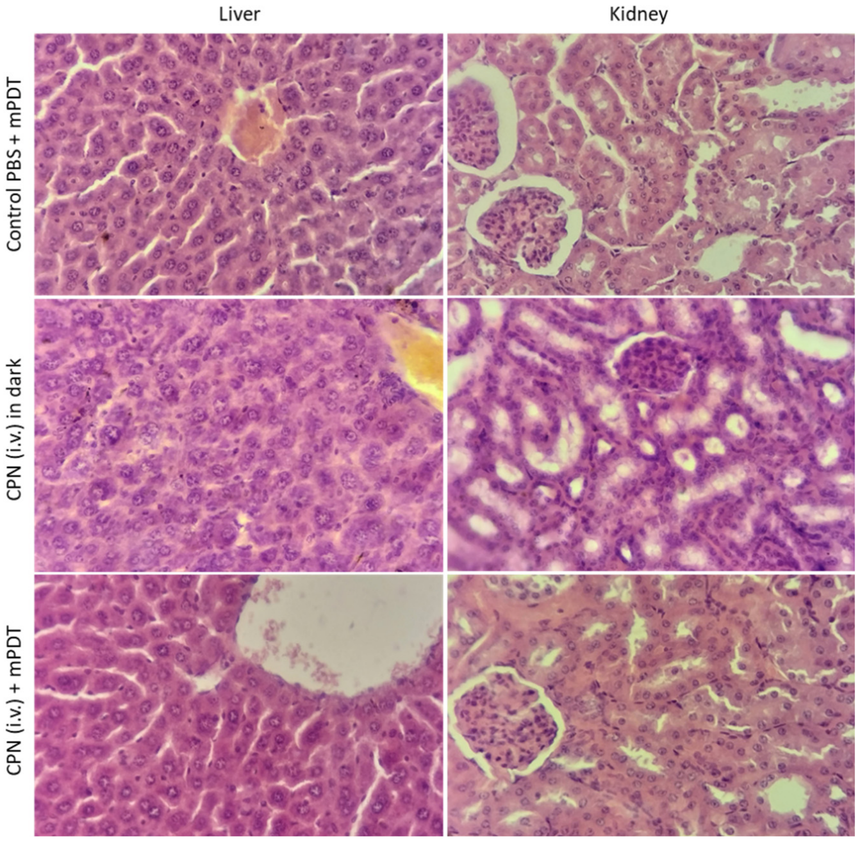

Figure S9. Histological evaluation of detoxifying organs after mPDT protocol. Representative histological images (40X) of organs such as liver and spleen from PBS injected and irradiated group, injected with CPNs (i.v.) and not irradiated and injected with CPNs (i.v.) and irradiated with mPDT. Organs were collected at day-12 time point after PDT treatment.
